# Plastic Valorization into Added-Value Products via Microwave and Conventional Pyrolysis: A Review

**DOI:** 10.1021/acsenvironau.5c00077

**Published:** 2026-01-06

**Authors:** Emmanuel Dan, Alan J. McCue, Davide Dionisi, Claudia Fernández Martín

**Affiliations:** † School of Engineering, Chemical Processes and Materials Engineering Group, University of Aberdeen, Aberdeen AB24 3UE, United Kingdom; ‡ Advanced Centre for Energy and Sustainability (ACES), Department of Chemistry, 1019 University of Aberdeen, Aberdeen AB24 3UE, United Kingdom

**Keywords:** Plastic waste, pyrolysis, microwave-assisted pyrolysis, environmental impact, conventional pyrolysis, life cycle assessment, sustainability, product selectivity, catalysts

## Abstract

Plastics are indispensable due to their versatility and low cost, but their accumulation poses major environmental challenges. Conventional waste management methods like landfilling, incineration, and mechanical recycling are inadequate, spurring interest in advanced valorization techniques. Pyrolysis offers a pathway to convert plastic waste into value-added products; however, traditional pyrolysis is energy-intensive, requiring high temperatures and long reaction times. Microwave-assisted pyrolysis (MAPP) has emerged as a superior alternative, enabling rapid heating (up to 50 °C/min), lower temperatures (≤500 °C), and shorter reaction times (∼10 min). MAP improves energy efficiency, yield, and selectivity toward valuable fractions such as bitumen, toluene, xylene (BTX), and medium-chain olefins. This review uniquely compares conventional pyrolysis and MAP for plastic waste conversion, analyzing process fundamentals, reactor designs, catalyst innovations (hierarchical, metal-modified, regenerable types), and product outcomes. Life cycle assessment data reveal MAP’s lower greenhouse gas emissions and water use compared to conventional pyrolysis, landfilling, and incineration. This work also highlights emerging applications, including hydrogen, jet fuel analogues, carbon nanotubes, and CO_2_ adsorbents from char, especially from underexplored plastics like PET, PS, and PVC. This work also systematically compares literature data across multiple studies, presenting the results in tabular form, while the identified gaps in catalyst standardization and product optimization delineate important directions for future research.

## Graphical abstract

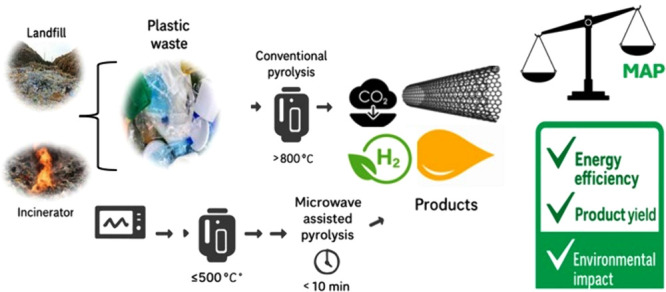

## 1 Introduction

Plastics have profoundly transformed modern life, driving innovations in medicine, transportation, communication, and technology.^1^ Their unique combination of versatility, durability, and low-cost continue to sustain their dominance over competing alternative materials, hence leading to a steady rise in the annual global plastic production (AGPP) over the past seven decades.^1^ AGPP is estimated to reach 1.1 billion tons by 2050 to meet growing demand.^2^

The extensive use of plastics has, however, led to an alarming rise in plastic waste generation. Approximately 381 million tons of plastic waste are produced annually, a figure expected to double by 2034.^3^ Less than 15% is recycled, while the remainder accumulates in landfills or natural ecosystems. If current trends persist, oceans could contain more plastic than fish by weight by 2050.^4^ Plastics’ non-biodegradable nature exacerbates this crisis, as they fragment through photo-oxidation and thermo-oxidation into harmful micro- and nanoparticles such as carbonyl species (ROO^•^) and macroradicals from the breakdown of hydroperoxides^5^ posing serious environmental risks.

Conventional plastic waste management methods such as landfilling, mechanical recycling, and incineration have proven inadequate in effectively addressing plastic pollution due to their environmental and economic limitations. Consequently, pyrolysis has emerged as a promising alternative for converting plastic waste into valuable products. This thermochemical process decomposes materials under nonoxidative conditions, with or without catalysts, and has shown success in valorising waste biomass,^6^ sewage sludge,^7^ and industrial waste.^8^ In the context of plastic waste management, pyrolysis enables the conversion of single and mixed plastics into hydrogen, liquid fuels, functional chemicals and carbon-based materials such as carbon nanotubes (CNTs) and activated carbons (ACs).^9^

Among pyrolysis methods, conventional and microwave-assisted pyrolysis are the most widely studied. Conventional pyrolysis has been scaled up in countries such as Japan, the USA, and India, but remains limited by high energy consumption, prolonged reaction times, and associated sustainability concerns. Microwave-assisted pyrolysis offers an alternative approach through volumetric heating, where electromagnetic energy is absorbed and converted to heat within the material, enabling rapid and selective heating.^10^ This intrinsic advantage enhances efficiency and product selectivity compared to conventional pyrolysis methods. Since its first report in 2001 by Ludlow-Palafox and Chase on high-density polyethylene (HDPE) pellets and toothpaste packaging materials,^11^ microwave-assisted pyrolysis has been extensively studied for various plastics such as polyethylene (PE), polypropylene (PP), polystyrene (PS), polyvinyl chloride (PVC), and polyethylene terephthalate (PET) with promising outcomes.^12–15^ Despite growing research, most reviews to date have focused on isolated aspects such as liquid fuel production, reactor design, or sustainability considerations. However, comprehensive comparative analyses of conventional and microwave pyrolysis, particularly regarding environmental performance and the valorization potential of derived products, remain limited.^16–22^

Consequently, this review presents a unique and comprehensive comparison of both conventional and microwave-assisted pyrolysis of plastic waste—an approach that distinctly sets it apart from previous reviews which predominantly emphasize on microwave techniques. It offers an in-depth analysis of process fundamentals, reactor configurations, operating parameters, catalyst types used and their effects, and comparative product yields. A key novelty lies in the inclusion of LCA, providing valuable insights into the environmental trade-offs between the two methods—an aspect largely absents or briefly mentioned in earlier literature. Furthermore, this review extensively explores the value-added applications of all product fractions (liquids, gases, and char) obtained, including CO_2_ adsorbents, hydrogen, jet fuel analogues, and CNTs, highlighting both their end-use potential and economic relevance. It features detailed tabular summaries of experimental data from studies involving single, mixed, and copyrolyzed feedstocks, enabling reproducibility and supporting future meta-analyses. Additionally, it identifies critical research gaps such as the need for standardized catalytic studies and the underexplored potential of plastic-derived char for CO_2_ capture application. Importantly, this review consolidates recent developments in CO_2_ adsorbent using plastic-derived adsorbents, including PET, PS, PVC, and mixed plastic waste—an area that remains underrepresented in existing literature and adds significant value to current research data.

This work is intended to serve as a valuable resource for both researchers and practitioners by summarizing the state-of-the-art developments, identifying pressing challenges, and guiding future innovations for the sustainable conversion of plastic waste into value-added products

## 2 Plastic Waste Recycling via Thermochemical Conversion: The Benefits of Pyrolysis

Utilizing plastic waste as a feedstock for producing value-added products offers a promising solution for sustainable waste management. Conventional plastic waste treatments include incineration, pyrolysis, gasification, and mechanical recycling, while emerging technologies such as plasma treatment, sub- and supercritical water gasification, and photoreforming show potential.^23^ However, these newer methods face scalability and economic issues. Plasma-assisted processes, despite their efficiency, suffer from high costs, unstable control, and operational complexity.^24^ Sub- and supercritical water treatments offer advantages such as high solubility, and rapid reaction rates,^25,26^ but are constrained by the need for extensive high-pressure systems, high energy consumption, and safety concerns.^27^ Table S1 shows some of the conventional plastic waste recycling methods such as mechanical, chemical, thermal, and thermochemical processes and summarizes their key advantages and limitations.

Mechanical recycling has reduced landfill accumulation but remain limited, as only certain polymers such as PE and PET are commonly recycled.^28^ The low recyclability of plastics like PS and PVC,^29,30^ and contamination issues further restrict efficiency.^31^ Thermal methods, such as incineration, enable energy recovery, but generate toxic emissions, including polychlorinated dibenzofurans, benzene, acetaldehyde, phosgene, hydrochloric acid, and polychlorinated dibenzo-dioxins, contributing to air pollution and public health concerns^32^ as well as polycyclic aromatic hydrocarbons (PAHs), CO, volatile organic compounds (VOCs), and carbon particulates from polyolefins.^33^

Thermochemical recycling methods such as pyrolysis, gasification, and hydrothermal processing are well-established techniques for converting plastic waste into valuable products.^34^ However, their high-temperature requirement increases energy and operational cost. As shown in Figure 1, each process differs in operational conditions and product recovery. Pyrolysis stands out for its process simplicity and relatively lower operating temperatures (300–700 °C), depending on the desired product, whereas gasification requires higher temperatures (500–1300 °C) and gasifying agents such as steam, oxygen, or air.^35^ Hydrothermal processing operates at lower temperatures but demands extreme high pressures of up to 200 bar and a water medium.^36^ The added complexity and safety risks of gasification and hydrothermal methods makes them more energy-intensive and environmentally burdensome than pyrolysis.

**Figure 1:**
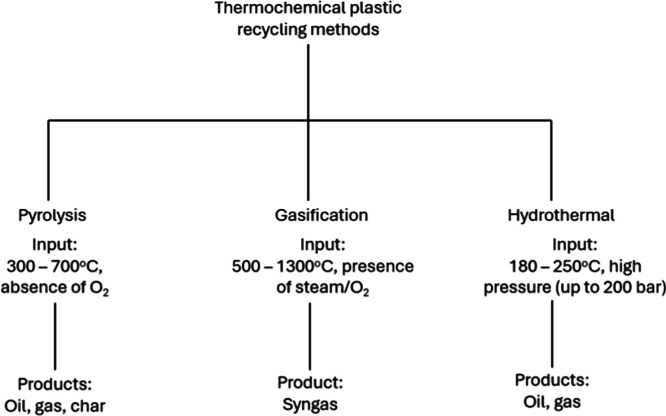
Thermochemical plastic recycling methods.

Pyrolysis is the most promising plastic recycling route due to its operational flexibility, cost-effectiveness, and ability to process various polymers, including polyolefins, PS, PET, and mixed plastics.^37^ It converts plastic waste into liquid oil, gas, and char, which can serve as fuels or chemical feedstocks.^37^ Unlike mechanical recycling, which requires clean and sorted inputs, pyrolysis accommodates mixed and contaminated plastics, reducing waste volume and pollution.^38^ It typically operates at atmospheric or slightly elevated pressures and moderate temperatures (500–600 °C), balancing energy efficiency with high-value output.

Co-pyrolysis allows simultaneous treatment of plastics with other wastes, such as biomass,^39^ and integration with processes like anaerobic digestion^40^ further expands its applicability. Although pyrolysis can emit acidic gases, nitrogen oxides (NO_*x*_), VOCs, and CO_2_,^41,42^ these can be effectively mitigated through established abatement systems, including calcium hydroxide neutralization, catalytic reduction, amine scrubbing, and activated carbon adsorption.^43^

Life cycle assessments show pyrolysis generally has a lower environmental footprint than incineration or landfilling. For example, pyrolysing PE for ethylene recovery proved cost-effective (€0.386/kg vs €0.835/kg for naphtha-derived ethylene) while reducing human health, ecosystem, and resource impacts by up to 164%.^44^ However, results can vary with context and methodological parameters as demonstrated in the studies reported by Demetrious and co.^45^

Other LCA studies reinforce the environmental benefits of pyrolysis. Dong et al.^46^ reported that among various waste-to-energy (WtE) technologies, pyrolysis achieved up to 68% lower greenhouse gas (GHG) emissions than the EU grid mix when energy recovery systems were optimized. Faraca et al.^47^ found that pyrolysis outperformed mechanical recycling in global warming potential (374 vs 940 kg CO_2_ equiv/tonne), particularly under low sorting efficiencies. The environmental performance further improves when pyrolysis products replace carbon-intensive materials such as paraffin or naphtha.

From an economic perspective, pyrolysis also offers significant benefits. Benavides et al.^48^ showed that converting non-recycled plastics into ultra-low sulfur diesel (ULSD) reduced GHG emissions by up to 14%, fossil fuel use by 83%, and water consumption by 58% compared to conventional diesel. When used instead of standard waste management routes, pyrolysis achieved a net GHG reduction of 0.60 tCO_2_e per tonne of plastic, while co-products such as char, fuel gas, and naphtha enhanced overall sustainability. Hernandez et al.^49^ reported that LDPE pyrolysis to lubricants yielded a high internal rate of return (IRR > 17%), outperforming gasification, hydrocracking, and hydrothermal liquefaction. Although hydrogenolysis and hydrocracking had slightly lower emissions, pyrolysis provided a better balance between environmental impact and profitability, especially with optimizations such as nitrogen recycling. Volk et al.^50^ further demonstrated that combining mechanical recycling with pyrolysis of residuals was more cost-effective (€0.14/kg input) and achieved a 16% improvement in carbon efficiency, along with substantial energy and GHG reductions. Cappello et al.^51^ also showed that converting HDPE to high-quality lubricants achieved production costs as low as $0.60/kg and market values between $1.56 and $3.12/kg, offering up to 25 times higher profit per ton of HDPE compared to traditional material recovery facilities. The process also reduced CO_2_ emissions by up to 52% relative to petroleum-based lubricants and 74% relative to polyalphaolefin (PAO) lubricants. In summary, techno-economic and life cycle studies underscore the potential of pyrolysis as a sustainable and profitable route for plastic waste management. When optimized, it reduces environmental burdens while yielding valuable products, supporting circular economy strategies and mitigating the global plastic waste crisis.

Despite these advantages, several technical and operational challenges remain. Catalytic pyrolysis, which enhances selectivity toward aromatics and light hydrocarbons, is limited by catalyst deactivation due to coke deposition and poisoning.^52^ Coke forms when heavy hydrocarbons and aromatics deposit carbon residues on catalyst surfaces, blocking active sites and reducing porosity. Contaminants such as chlorine from PVC, sulfur, and heavy metals further exacerbate deactivation by binding irreversibly to catalytic sites.^53^

These challenges are intensified by the heterogeneity of mixed plastic waste. To mitigate this, recent studies have developed hierarchically structured and metal-modified catalysts such as Ni-ZSM-5, which combine micropores for shape selectivity with mesopores that enhance molecular diffusion, improving coke resistance and product selectivity.^46,54^ Regenerable catalysts also offer a promising approach, allowing periodic oxidative regeneration to remove coke and restore catalytic activity, thereby extending catalyst life and improving process sustainability.^55,56^

Another key challenge lies in minimizing undesirable byproducts such as PAHs, tar, and waxes formed at high temperatures or under non-optimized conditions.^57–60^ Maintaining moderate pyrolysis temperatures (450–550 °C) and short vapor residence times can reduce these side reactions.^52^ Additionally, selective catalysts like ZSM-5—especially metal-modified or hierarchically structured variants can direct the reaction pathway toward the formation of desirable products such as light olefins and aromatics, thereby reducing the formation of heavier or more reactive compounds.^61^

Finally, achieving stable control over product distribution, oil, gas, and char remains complex, as incomplete pyrolysis of polyolefins like PE and PP can yield viscous oils requiring further upgrading to meet fuel standards.^62^

## 3 Conventional Method of Plastic Waste Pyrolysis

Pyrolysis, first reported in 1862 for identifying isoprene as a pyrolytic product of rubber,^63^ involves thermal decomposition of materials in an inert atmosphere. In plastic pyrolysis, polymers are heated in the absence of oxygen, breaking long chains into smaller molecules. Heat transfer occurs through conduction, convection, and radiation in reactors such as fixed-bed, rotary-kiln, and fluidized-bed systems, selected based on feedstock type, desired uniformity, scalability, and cost.^64^

The process is governed by key operating parameters—temperature, heating rate, and residence time resulting in slow, fast, and flash pyrolysis modes. Slow pyrolysis operates at 500–700 °C with low heating rates, fast pyrolysis at 850–1200 °C with shorter durations, and flash pyrolysis at even higher temperatures with very rapid heating. These modes yield different product distributions like gas, liquid (oil), and solid (char), depending on process conditions and plastic type.^65^ For instance, pyrolysis at 800 °C and a 350 °C/min heating rate in a fixed-bed reactor under nitrogen produced the product yields shown in Figure 2.^66^ Results for PP and mixed plastics (45% PP, 35% LDPE, 25% HDPE) at 450 °C are reported elsewhere.^67,68^ Typical polyolefin pyrolysis yields range from 2–47% gas, 0–16% char, and 31–91% oil.^69^

**Figure 2:**
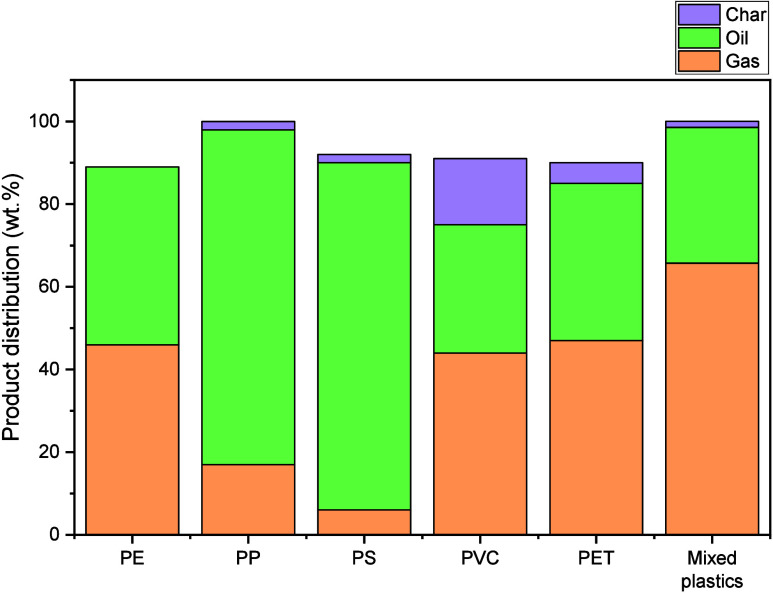
Yield of products from plastic pyrolysis.

The solid fraction is generally minimal because plastics contain high volatile matter (>99 wt %) and very low fixed carbon (0–0.22 wt %).^9,70^ PET, however, generates more char (>91 wt % C),^71^ which serves as an excellent precursor for CO_2_ adsorbents. Chars from PS and PVC pyrolysis have also been utilised as adsorbents for CO_2_ adsorption, though cross-linking agents are often required to improve yield and performance. In PVC pyrolysis, a precarbonization dechlorination step is necessary to avoid toxic chlorinated byproducts.^72^ Recently, adsorbents from mixed plastic-derived chars have been reported as efficient adsorbent precursors.^73,74^

Plastic-derived adsorbents show variable CO_2_ adsorption capacities, ranging from 0.003 mmol/g to 3.87 mmol/g at 25 °C and 1 bar,^75^ depending on activation conditions such as temperature, duration, and activating agents. Nitrogen doping using urea, organically modified montmorillonite (OMMT), or carbazole enhances CO_2_ capture through increased surface basicity.^75^ Conversely, producing adsorbents from PVC and PS requires additional cross-linking agents such as 4,4′-bis­(chloromethyl)-1,1′-biphenyl, α, α′-dichloro-*p*-xylene, and formaldehyde dimethyl acetal as shown in Table S2, which raises process complexity and cost.

### 3.1 Conventional Catalytic Pyrolysis

Catalytic pyrolysis enhances the yield and quality of pyrolysis products by introducing catalysts that promote selective reactions and reduce energy consumption. Catalysts may be incorporated in a one-step process via direct mixing with plastics—or in a two-stage configuration, where plastics are first thermally decomposed (∼500 °C), and the resulting volatiles are passed to a second catalyst-loaded reactor (700–900 °C). Direct mixing at 450–500 °C^76^ has been reported but is often hindered by catalyst deactivation due to coke formation or contamination by impurities.^77^

The use of catalysts improves liquid oil quality,^78^ minimizes the formation of undesired byproducts,^79,40^ lowers energy requirements, and enable selective bond cleavage to maximize product yield.^80^ The catalytic pyrolysis of polyolefins proceeds through sequential reactions including chain scission, isomerization, oligomerization, hydrogen transfer, and aromatization.^77^ Figure 3 illustrates the mechanism of CNT formation from polyolefins over Fe/Al_2_O_3_ catalysts.^81^

**Figure 3:**
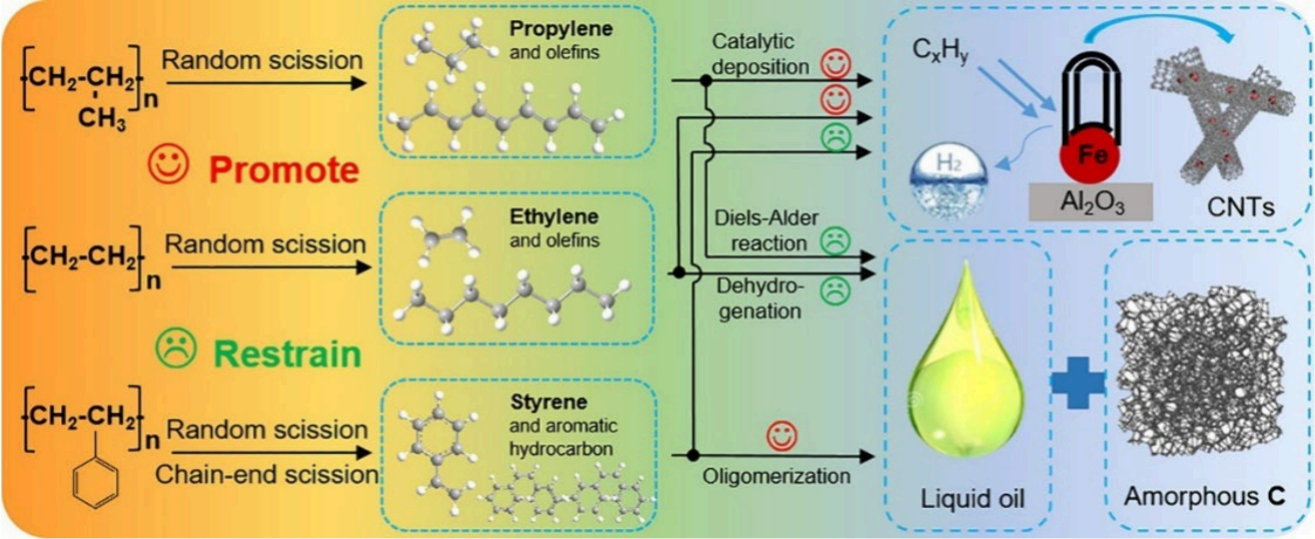
Mechanism of catalytic plastic pyrolysis of plastics. Reproduced with permission from ref 81. Copyright 2021 Elsevier.

Polyolefins such as PE and PP undergo random C–C scission forming short-chain intermediates that crack further to methane and ethylene via β-scission.^82^ The vapors interact with Fe catalysts, leading to Fe_3_C formation and CNT growth. Increasing Fe_3_C crystal size produces thicker CNTs,^83^ but excessive particle growth reduces carbon solubility and limits CNT formation.^84,85^ PS degradation primarily yields aromatic hydrocarbons, such as styrene, through random and chain-end scission.

Table S3 summarizes catalytic pyrolysis studies for LDPE, HDPE, PP, and PS. PVC pyrolysis is less explored due to low liquid yields 87.7 wt % gas, 12.3 wt % liquid at 500 °C with no char,^86^ and the release of corrosive chlorinated hydrocarbons.^86,87^ Pre-pyrolysis dechlorination via hydrothermal ammonia treatment^88^ or near-critical methanol (NCM) solution^89^ mitigates these issues. Catalysts employed in plastic pyrolysis include Ni, Fe, ferrocene, Co, Cu, zeolites, bimetallic (Ni–Mn, Ni–Fe, Co–Mo) and trimetallic catalysts (Ni-X-Al, where X = Zn, Mg, Ca, and Ce) system.

Nickel-based catalysts are particularly effective for hydrogen generation. Ni–Mn/Al_2_O_3_ and Ni/Al_2_O_3_ enhance H_2_ production at >800 °C, achieving yields up to 183.65 mmol/g during HDPE pyrolysis at 900 °C in a conical spouted bed reactor.^90^ The superior performance arises from nickel’s dehydrogenation and reforming activity. Metal–supported catalysts also promote solid carbon formation. For instance, PS pyrolysis over Fe/MgO yielded 71.3 wt % multiwalled CNTs in a tubular fixed-bed reactor.^91^ Liquid fractions rich in aromatics are also valuable; Co/MgO and natural zeolites achieved high oil yields (62.8 wt % for LDPE, 83.5 wt % for PS), while zeolites such as ZSM-5 and β-zeolite effectively increase aromatic and light hydrocarbon formation.

Catalyst composition and reaction parameters determine product selectivity—Ni and Fe catalysts favor CNT and H_2_ production, whereas zeolitic catalysts favor liquid oil and light hydrocarbon yields. Transition metals (Ni, Fe, Ni–Fe) facilitate cracking and reforming of vapor-phase intermediates, generating CNTs and hydrogen via carbon–hydrogen bond cleavage.^92,93^ Steam addition enhances hydrogen yield through reforming and coke reduction but can modify CNT morphology.^94^ Polyolefins (PE, PP) are ideal feedstocks due to their high C/H ratio, while halogenated plastics like PVC can poison active sites.^9^ In contrast, zeolite catalysts (e.g., ZSM-5, Ni-ZSM-5) operated at 450–550 °C with short vapor residence times (<5 min) maximize liquid oil yield.^95^ Absence of steam and controlled catalyst-to-feed ratios further favor liquid over solid carbon formation. Thus, tailoring catalyst type and thermal conditions allows precise control of pyrolysis outputs for specific valorization goals.

Catalyst acidity and pore structure strongly influence yield and selectivity. Brønsted acid sites enhance C–C cracking and aromatization, while excessive acidity accelerates coke deposition and deactivation. Balancing acid strength and density is therefore crucial for sustained activity and high-quality product formation.^96^ Pore architecture also determines catalyst effectiveness: microporous zeolites like ZSM-5 and H-β offer high shape selectivity for light hydrocarbons but limit diffusion of bulky intermediates. Mesoporous materials such as SBA-15 and Al-SBA-15 alleviate diffusion constraints and enhance conversion efficiency, especially for large polymers like LDPE. Bimodal or hierarchical zeolites (e.g., desilicated ZSM-5) combine micro- and mesopores, improving surface accessibility and reducing coke buildup. These catalysts enhance aromatic yields while maintaining stability.^97^ Catalytic performance depends on matching catalyst properties to plastic type and desired output. Agulló et al.^98^ observed that while H-Ferrierite exhibited high acidity, limited pore access restricted performance, whereas H-β with moderate acidity and larger pores displayed superior activity. Similarly, Tian et al.^99^ reported that HZSM-5, with optimal acid density and pore size, produced the highest monocyclic aromatic selectivity during LDPE pyrolysis. Onwudili et al.^100^ demonstrated that ZSM-5 and Y-zeolite catalysts enhanced C_5_–C_15_ and aromatic yields from mixed plastics, with Y-zeolite favoring benzene and toluene formation due to stronger acidity, while Al_2_O_3_ promoted gaseous products such as H_2_ and C_1_–C_4_ hydrocarbons. Zhang et al.^101^ showed that spent Y-zeolite, after modification to reduce acidity, increased gasoline-range hydrocarbons to 52% and reduced coke formation through olefin isomerization. Hierarchically structured HZSM-5 developed by He et al.^102^ produced up to 92 wt % combined light olefins and monocyclic aromatics during PP pyrolysis.

Conventional catalytic pyrolysis using traditional reactors demonstrates high potential for plastic waste valorization, though its environmental implications require attention. Alongside valuable products, the process emits acidic gases, NO_*x*_, PAHs, VOCs (e.g., ethylene, propylene), and CO_2_, contributing to air pollution and climate impacts.^41^ LCA indicate that pyrolysis of PE and PP generates approximately 2,687 kg CO_2_ equiv/t of feedstock,^103^ like 2,558 kg CO_2_ equiv/t reported for HDPE.^104^

Nevertheless, LCAs consistently show pyrolysis to have lower environmental impacts than incineration and comparable or superior performance to mechanical recycling. Pyrolysis of mixed plastics exhibits roughly 50% lower climate change impact and life cycle energy use relative to energy recovery options, and a much smaller carbon footprint (−0.45 t CO_2_ equiv/t vs 1.89 t CO_2_ equiv/t) compared with virgin production.^41^ Reported global warming potential (GWP) varies with product type, ranging from – 1.46 kg CO_2_ equiv for HDPE-derived gaseous fuels to – 0.44 kg CO_2_ equiv for PET-derived liquid fuels.^105^

Integrating value-added carbon material synthesis can further reduce environmental burdens. PET pyrolysis co-producing pyrolysis oil and multiwalled CNTs achieved negative GWP due to the avoided impacts of CNT production. This integrated process also exhibited minimal eutrophication (−0.145 kg P equiv freshwater; – 0.014 kg N equiv marine) and terrestrial acidification (−2.29 kg SO_2_ equiv), with negligible contributions to particulate formation, ozone depletion, and land use.^106^ Conversely, PET conversion to diesel or cyclo-hexanedimethanol showed GWP values from 0.35 to 3.77 kg CO_2_ equiv, depending on process configuration.^105^

While detailed LCA analyses of catalytic pyrolysis of plastics are beyond the present scope, readers are encouraged to consult prior reviews^107,108^ for in-depth evaluations of environmental methodologies and impact categories for conventional plastic pyrolysis

## 4 Microwave-Assisted Pyrolysis of Plastics

Conventional plastic pyrolysis relies on external heating through conduction, convection, and radiation, where heat transfers from a furnace coil to the sample surface before diffusing inward. This indirect mechanism causes inefficient and non-uniform heating, requiring longer residence times and higher energy input.^109^ Microwave heating, in contrast, generates heat volumetrically as microwave radiation penetrates and is absorbed by the material, producing higher temperatures at the core than at the surface. This minimizes heat loss and enables rapid, selective heating.^109^ Unlike conventional heating, microwave energy absorption is less dependent on thermal conductivity or specific heat capacity,^110^ resulting in faster and more energy-efficient pyrolysis. The physics of microwave heating are detailed elsewhere.^111^

Microwave-assisted pyrolysis has been widely demonstrated for converting diverse wastes into high-value products such as fuels, adsorbents, and chemicals starting from scrap tires,^112^ automotive engine oil,^113^ sewage sludge,^114^ electrical/electronic board plastic waste,^115^ and biomass sources like rice straw,^116^ coffee hulls,^114^ microalgae,^117^ wool,^118^ waste tea,^119^ and wheat straw.^120^

Ludlow-Palafox and Chase examined microwave pyrolysis of HDPE and laminated materials at 500–700 °C using a modified 5 kW microwave oven, producing high-quality hydrocarbons and nearly 100% recovery of reusable aluminum.^11^ The products consisted mainly of linear alkenes and alkanes, with minimal byproducts, demonstrating the method’s scalability and energy efficiency. Subsequent studies confirmed the successful application of microwave-assisted pyrolysis to PP,^12^ PS,^54,121^ PVC,^122^ LDPE,^123^ and mixed plastics.^15,14^

Like conventional pyrolysis, microwave-assisted pyrolysis (MAP) yields liquid (oil), gas, and solid char. The char, composed mainly of carbon (58.1–72.9 wt %),^124^ can serve as a precursor for adsorbents or energy storage materials such as supercapacitors. Nitrogen functionalization (e.g., with urea) enhances its CO_2_ adsorption.^75^ While microwave carbonization of plastic for char production and subsequent activation for adsorbents remains largely unexplored, recent studies have investigated microwave activation of char from conventional plastic pyrolysis for CO_2_ adsorption.^71,73^

A simplified schematic of a MAP setup is shown in Figure 4, where vapors generated under microwave irradiation are condensed into liquid and gas fractions. Analytical tools such as gas chromatography with mass spectrometry (GC-MS) and Fourier Transform Infrared Spectroscopy (FTIR) can be integrated for real-time monitoring, analysis, and optimization of the process

**Figure 4:**
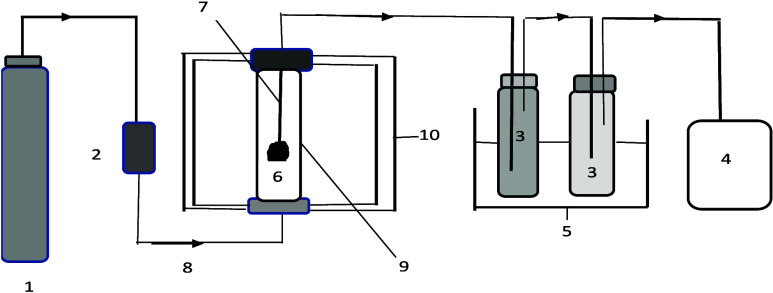
Schematic diagram of a microwave setup: 1. N_2_ gas; 2. Gas flow meter; 3. Lowboiling solvent; 4. Gas bag; 5. Ice bath; 6. Sample; 7. Thermocouple; 8. Gas inlet; 9. Glass tube reactor; 10. Microwave cavity.

A key limitation of MAPP lies in plastics’ poor microwave absorption. Polyolefins are nearly transparent to microwaves, exhibiting very low loss tangent values (tan δ = 0.0001–0.005), far below those of efficient microwave susceptors like silicon carbide, SiC (0.37),^125^ activated carbon (0.31–0.9),^126^ and graphite (0.36–0.67).^127^ Hence, susceptors are added to promote rapid and uniform heating.

Two main heating approaches exist: pre-mixed and non-premixed.^128^ In the pre-mixed method, plastics are blended with powdered or pelletized susceptors before irradiation; in the non-premixed method, the susceptor is pre-heated and plastics are added gradually. Uniform particle size and distribution are critical to prevent uneven heating. Pre-melting the plastic, mixing with powdered susceptors, and cooling before microwave treatment offers improved homogeneity and temperature control.^124^

Microwave-assisted pyrolysis may be conducted with or without catalysts. In catalytic MAPP, catalysts modify reaction pathways, enhance selectivity, and reduce energy requirements.^129^ Certain susceptors, particularly metals and metal oxides also act as catalysts, generating localized heating and promoting reactions such as cracking, dehydrogenation, and isomerization. For example, Jie et al. achieved one-step microwave depolymerization of HDPE using FeAlOx, which functioned simultaneously as a microwave absorber and catalyst.^15^

Tables S4–S6 summarize major studies on MAPP, including single, mixed, and co-pyrolysis systems. These studies detail reaction conditions, catalyst and susceptor types, feedstock modifications, and product yields. MAPP has been widely explored for single polymers such as PS, LDPE, PVC, PP, and HDPE, as well as mixed plastics like PP/PS, typically under inert N_2_ atmospheres. Recent work also includes microwave co-pyrolysis of plastics with biomass-derived materials such as sugar cane bagasse, rice straw, lignin, bamboo, wheat straw, coconut shells, microalgae, tires, and chili straw, aiming to enhance product yield and oil quality. Various catalysts and susceptors—HZSM-5, SiC, carbon, graphite, ACs, fly ash, aluminum, and HNO_3_—have been used to influence product selectivity. Notably, HZSM-5 enhances oil yield and quality, while graphite and ACs favor aromatic hydrocarbon production.

Oil fractions typically dominate MAPP outputs. For example, PS treated with carbon susceptors yielded 92.3 wt % oil,^130^ and PP pyrolysed with graphite and AC produced 48–69 wt % liquid.^123^ LDPE copyrolyzed with sugar cane bagasse and HZSM-5 yielded 43.48 wt % bio-oil rich in alkenes and oxygenates. Gas yields vary, with higher gas fractions often linked to hydrogen and light hydrocarbon formation, while solid yields remain low (<20%).^131,132^ Oils from microwave pyrolysis of PS and PP are particularly aromatic, containing styrene, benzene, and naphthalene.^121^

Co-pyrolysis of plastics with biomass enhances oil quality due to synergistic hydrogen transfer between hydrogen-rich plastics and oxygen-rich biomass. This interaction promotes deoxygenation reactions such as decarboxylation, decarbonylation, and dehydration reducing oxygen content and increasing oil heating value, stability, and fuel suitability.^133^ The complementary composition of plastics (high H/C, low O) and biomass (low H/C, high O) facilitates in situ hydrogen donation, improving oil energy density and hydrocarbon content compared to single-feed pyrolysis.

Among plastics, PS and PP show the highest oil yields, while LDPE and HDPE yield more balanced solid, liquid, and gas fractions. Catalytic MAPP of LDPE has been extensively studied to optimize fuel-range hydrocarbons. Ding et al. employed NiO and HY zeolite catalysts at 450–600 °C, achieving up to 56.53 wt % oil with HY (LDPE: HY = 5:1). Incorporating NiO (HY: NiO = 15:1 to 3:1) further improved aromatic hydrocarbon content and fuel quality.^129^ Zhang et al. found that LDPE pyrolysis with ZSM-5 produced 88.49% mono-ring aromatics and 32.58 wt % upgraded oil at 450 °C and a 2:1 reactant-to-catalyst ratio, due to ZSM-5′s strong acidity promoting aromatization.^134^ Juliastuti et al. also reported 28.12 wt % oil from LDPE with natural zeolite (300–550 °C, 45–90 min).^135^ Transition metal-based catalysts have been explored for gas and carbon nanomaterial co-production. Zhang et al. demonstrated that Fe-based catalysts doped with Co, Ni, and Cu yielded significant hydrogen and carbon nanotubes. The Co_1_Fe_1_Ox catalyst produced 60.7 mmol H_2_/g plastic, while Co_1_Fe_9_Ox achieved 63.64 mmol/g.^136^ Microwave pyrolysis of mixed plastics using ZSM-5 at 620 °C for 90 s achieved 48.9 wt % liquid with enhanced gasoline-range hydrocarbons.^69^

Suriapparao et al. studied catalytic copyrolysis of PS and PP with rice straw and sugar cane bagasse using HZSM-5, achieving 82 and 98 wt % oil yields, respectively. The copyrolyzed bio-oil resembled light fuel oil (calorific value 43 MJ/kg, viscosity 1 cP, density 0.850 g/cm^3^, flash point 70 °C), dominated by C_8_–C_20_ hydrocarbons. PS–biomass mixtures yielded styrene-rich oils containing ethylbenzene and cumene. However, due to compositional complexity, direct oil utilization requires further upgrading.^133^ Char characterization from MAPP reveals variations in carbon content and higher heating value (HHV). Pure plastic-derived chars exhibit high carbon content (92–95 wt %) and HHVs (32–33 MJ/kg). Co-pyrolysis with biomass slightly reduces these values; bagasse–PP and bagasse–PS chars contained 84.5 and 90.2 wt % carbon with HHVs around 29 MJ/kg, whereas rice straw mixtures yielded lower carbon (71–72 wt %) and HHVs (∼25 MJ/kg) due to higher ash content. In all cases, biochar susceptors showed HHVs between 25–29 MJ/kg, indicating moderate energy potential.^133^

Catalytic upgrading of pyrolysis vapors has also shown promise. Fan et al. used ex-situ MgO during LDPE pyrolysis, shifting selectivity toward lighter hydrocarbons. Without a catalyst, 46.3 wt % oil and 52.8 wt % gas was produced, dominated by C_12_ + alkenes. Adding MgO (LDPE: MgO = 10:1) reduced oil to 30.3 wt % and increased gas yield to 67.0 wt %, with oils enriched in C_5_–C_12_ alkenes.^137^

Jie et al.^15^ reported a one-step catalytic depolymerization of real-world plastic waste including HDPE (milk containers), LDPE (plastic bags), PP (food wraps), and PS (plastic foam) using a composite FeAlOx catalyst that also served as a microwave susceptor. The catalyst was prepared by mixing iron nitrate, aluminum nitrate, and citric acid in distilled water. The mixture was then calcined at 350 °C for 3 h to form a powder. The microwave pyrolysis experiment was conducted at 1000 W microwave power for 3–5 min, gas chromatography revealed a hydrogen yield of 55.6 mmol/g plastic, with approximately 90 vol % H_2_ in the evolved gases. Additionally, carbon production reached consisting of over 92 wt % MWCNTs. This exceptional hydrogen recovery was attributed to the synergistic effects of microwave irradiation and the Fe-based catalyst. These findings highlight microwave catalytic pyrolysis as a promising solution to plastic pollution and a viable method for generating clean hydrogen fuel and value-added carbon products.

As shown in Tables S3–S5, product distribution in MAPP strongly depends on the plastic feedstock, catalyst, and susceptor used. Differences in product yields and compositions arise mainly from variations in the physicochemical properties of catalysts and susceptors specifically, their microwave absorption capacity, acidity, redox behavior, and surface characteristics. Suriapparao et al.,^123^ demonstrated this by pyrolyzing PP using various susceptors (graphite, AC, SiC, Al, and fly ash) under identical microwave power (0.45 kW). Graphite produced 48.16 wt % liquid and 50.66 wt % gas, while fly ash yielded 21.07 wt % liquid and 78.23 wt % gas. These differences were attributed to the susceptors’ distinct dielectric and thermal conductivities, which influenced heating uniformity and cracking intensity.

In a broader study, Suriapparao and Vinu^123^ compared ten susceptors, including graphite, AC, SiC, lignin, Al, and fly ash, during microwave pyrolysis of some synthetic polymers. Graphite achieved the highest oil yield (53 wt %) with abundant monoaromatics and 95% energy recovery, reflecting its excellent microwave coupling and catalytic cracking efficiency. Graphite and AC, with high conductivity and surface area, enabled uniform heating that promoted vapor cracking and deoxygenation. In contrast, Al and fly ash generated lower liquid yields due to poor microwave absorption and differing catalytic tendencies—Al favoring hydrogenation and SiC promoting dehydrogenation and cracking. The metal oxides (Fe, Al) in fly ash enhanced secondary cracking and gas formation through deoxygenation. Furthermore, susceptor loading and plastic–susceptor contact significantly affected heat transfer and product selectivity; Suriapparao and Garlapati^124^ showed that lower graphite loading and higher microwave power accelerated heating, increasing light hydrocarbon yields from PP.

Complementary findings were reported by Putra et al.^138^ who studied mixed plastics (PS, PP, LDPE) using Fe-based susceptors in powder and coil forms. A 10:5 iron powder-to-coil ratio produced up to 69.24 wt % oil, while other ratios shifted the balance toward gas and waxes. The enhanced oil yield was attributed to uniform heating by iron powder, whereas coils reflected microwaves, causing hot spots, metal agglomeration, and arc discharge that favored gas and char formation. Product distribution in MAPP depends not only on catalysts and susceptors but also on operational parameters such as temperature, catalyst loading, vapor residence time (i.e., the amount of time released gases spent in contact with catalyst within the reactor), feed composition, and heating rate.

Importantly, the product distribution during microwave-assisted pyrolysis does not depend solely on the choice of catalyst, susceptor, or their combination. Rather, key experimental parameters—such as temperature, catalysts-to-feedstock loading, vapor residence time (i.e., the amount of time released gases spent in contact with catalyst within the reactor), feedstock composition, and heating rate—play equally significant roles in determining the yield and selectivity of pyrolysis products. Ludlow-Palafox and Chase^11^ showed that increasing pyrolysis temperature from 500 to 700 °C during HDPE and aluminum/polymer laminate pyrolysis altered hydrocarbon profiles and volatile residence times. Similarly, Khaghanikavkani et al.^139^ demonstrated that uniform temperature control in a rotating SiC-based reactor achieved a high oil/wax yield (73%), emphasizing the importance of heat uniformity and residence time.

Other studies highlight synergistic effects between process conditions and catalyst properties. Suriapparao and Tejasvi^19^ found that particle size, feed type, and reactor configuration collectively influence product composition beyond catalyst effects. Li et al.^140^ achieved high hydrogen yields (44.07 mmol/g) and bamboo-shaped carbon nanotubes from polypropylene using Al–Fe catalysts, with gas yields up to 94.21 wt % due to microwave-induced oxygen vacancies that promoted secondary cracking and reforming. Similarly, Zeng et al.^141^ reported that BTX yields during copyrolysis of waste cooking oil and LDPE depended on catalyst type (HZSM-5 > HY > Hβ > SAPO-34), temperature (550 °C pyrolysis, 450 °C catalytic), and feed ratios. In the copyrolysis of plastics and algae, Suriapparao et al.^124^ observed that ZSM-5 enhanced cracking, producing more gas and coke, while biochar susceptors influenced energy distribution and heating uniformity, thus affecting yield and composition.

Overall, these studies demonstrate that MAPP outcomes result from a complex interplay between catalyst/susceptor properties and process parameters. Optimizing MAPP requires not only selecting suitable catalytic materials but also precisely controlling thermal conditions, feedstock characteristics, and reactor design to direct reactions toward desired products.

### 4.1 Microwave-Assisted Pyrolysis of Plastic under Reactive Atmosphere

Microwave pyrolysis of plastic has recently been explored under reactive atmospheres, particularly CO_2_, to improve product yield and purity.^142,143^ Despite its potential, studies employing CO_2_ to modify product distribution, increase syngas yield, and improve process efficiency remain limited. This represents a research gap, given the dual potential of such systems to valorise both plastic waste and greenhouse gases.

Compared with inert N_2_ systems, CO_2_-assisted MAP introduces gas-phase reactions that promote cracking and reforming. Under similar conditions, CO_2_ atmospheres typically yield higher syngas output, greater carbon conversion efficiency, and more diverse value-added products, whereas N_2_ favors stable but less reactive distributions.^143^ The presence of CO_2_ provides additional reaction pathways that facilitate the production of benzene, toluene, ethylbenzene, and xylenes (BTEX), olefins, hydrogen-rich syngas, light fuels, and even carbon nanomaterials.^144^

In CO_2_-rich environments, both thermal decomposition and chemical interactions occur. Acting as a mild oxidant, CO_2_ reacts with hydrocarbons and carbon residues through the reverse Boudouard and dry reforming reactions to generate CO and H_2_.^145,146^ These reactions enhance syngas energy content, suppress tar accumulation, and improve product quality. Mechanistically, a major difference between inert and reactive conditions lies in secondary reactions: PS pyrolysis under N_2_ mainly produces styrene and monocyclic aromatics, whereas under CO_2_, dehydrogenation and CO_2_-assisted reforming promote greater aromatic and syngas formation while reducing coke deposition.^146^

Zhang et al.^142^ developed a one-step microwave-driven CO_2_ gasification system for plastic waste, achieving high hydrogen and carbon conversion rates. Operating at 1000 W and 800 °C under 200 mL/min CO_2_, the AlNiOx catalyst (52.13 m^2^/g) produced up to 480 mmol H_2_ g^–1^ h^–1^, 70% carbon conversion, and 53% CO_2_ conversion, maintaining stability over five cycles. CO_2_ effectively enhanced carbon removal and syngas quality. Similarly, Jung et al.^147^ explored a two-stage fixed-bed MAP system using HDPE fishing net waste under 200 mL/min CO_2_ and 600 °C with 5 wt % Ni/SiO_2_ catalyst. The CO_2_-assisted system achieved H_2_ and CO yields of 1093 and 1157 mmol g^–1^ cat h^–1^, respectively, while suppressing coke formation and catalyst deactivation. Enhanced CO generation was attributed to the reverse water–gas shift reaction, allowing adjustment of H_2_/CO ratios. Luo et al.^143^ introduced a continuous microwave pyrolysis (CMP) process for LDPE integrating in situ CO_2_ reforming without solid catalysts. Operating between 450–650 °C with 0–100 vol % CO_2_, the process achieved a maximum gas yield of 96.44 wt % and syngas content of 66.61 vol %, with a tunable H_2_/CO ratio (0.65–2.88). Density functional theory (DFT) analysis indicated that CO_2_ facilitated oxidized carbon intermediates, enhancing carbon conversion.

Building on this work, Luo et al.^148^ optimized CO_2_-enhanced CMP of LDPE, identifying 25 vol % CO_2_ as optimal, achieving 81.69% CO_2_ utilization, 75.29% energy recovery, and syngas (H_2_ + CO) of 65.61 vol % with a flexible H_2_/CO ratio (0.62–3.99). Besides syngas, hydrogen and high-heating-value oils were also produced. This highlights the dual environmental benefit of plastic waste valorization and CO_2_ utilization, emphasizing the scale-up potential of continuous MAP for sustainable fuel and chemical production. However, CO_2_’s stability remains a limitation since it requires active reforming catalysts to become reactive. Without them, CO_2_ remains largely inert, reducing its contribution to syngas enhancement and tar suppression, thereby limiting process efficiency.^16^

### 4.2 Parametric Studies and Optimization in Microwave-Assisted Plastic Pyrolysis

The performance of MAPP depends on several operational parameters that determine product yield, composition, and distribution. Key factors include temperature,^149^ microwave input power (MIP),^150^ catalysts type,^141^ and their combinations.^151^ Although these parameters have been extensively studied, systematic comparisons under standardized microwave conditions across different plastic types remain limited, highlighting a gap in current research. This section focuses on the effects of the heating scheme and catalyst type, two of the most critical factors influencing MAPP efficiency and selectivity.

#### 4.2.1 Effect of Heating Scheme

The thermal degradation behavior of plastics dictates optimal pyrolysis conditions. Polymers such as PS and PVC decompose at lower temperatures due to weaker chemical bonds and lower molecular weights, whereas polyolefins like PE and PP, particularly HDPE require higher power inputs or temperatures owing to their crystallinity and strong C–C bonds.^152,37^ Microwave irradiation adds complexity through variations in dielectric properties, localized heating, and hot spots that significantly influence depolymerization. Therefore, aligning microwave power, particle size, and residence time with the specific degradation characteristics of each polymer is essential for maximizing yield and product quality.

For PS, a polymer with relatively low thermal stability, readily depolymerizes at moderate microwave power. Bartoli et al.^130^ reported 92.3 wt % liquid yield at 3 kW with minimal residue, while Undri et al.^13^ obtained efficient depolymerization to styrene-based oil at 1.2–3.0 kW. In contrast, PVC exhibits more complex degradation due to HCl evolution. Dai et al.^122^ found that increasing microwave power from 0.8 to 1.0 kW enhanced oil and gas yields while reducing char formation, indicating that sufficient energy input is crucial to drive secondary reactions.

Higher molecular weight plastics show more energy-intensive degradation. For LDPE, Backstrom et al.^151^ demonstrated that longer residence time and small temperature increases (170–180 °C) promoted long-chain dicarboxylic acid formation. Fan et al.^153^ observed that raising pyrolysis temperature from 450 to 600 °C during copyrolysis with lignin improved oil and gas yields by enhancing secondary cracking. HDPE, highly crystalline and thermally stable, required power above 6 kW for complete decomposition, yielding 37 wt % liquid.^13^ Similarly, PP exhibited limited breakdown even at higher power, remaining viscous without additional catalytic support.^132^ Condensation polymers like PET behave differently due to their ester linkages and high melting points. Liu et al.^154^ reported that PET pyrolysis using SiC at 500 W produced only gas and solid residues, dominated by benzoic acid derivatives, indicating incomplete decomposition.

Heating parameters are also critical when processing mixed plastics or cofeeding with biomass or oils. Wan Mahari et al.^155^ achieved an optimal liquid yield of 81 wt % during HDPE copyrolysis with waste frying oil at 550 °C, though excessive cracking at 600 °C increased gas and char yields. Similarly, Wu et al.^94^ and Zhang et al.^134^ found that higher microwave power and rapid heating promoted H_2_ and CH_4_ formation while suppressing solid residue. Overall, each polymer exhibits distinct thermal degradation behavior under microwave conditions. Effective process optimization requires adjusting microwave parameters—temperature, power, and residence time—to match the chemical and physical properties of each plastic. Understanding these interactions is essential for enhancing process efficiency, oil quality, and overall conversion performance in MAPP.

#### 4.2.2 Effect of Catalyst

Catalysts play a paramount role in MAPP, by reducing reaction temperature, improving selectivity, and accelerating conversion through enhanced free radical activity.^156^ Molecular sieve catalysts such as SAPO-34, ZSM-5, HY, Hβ, and MCM-41 are commonly employed due to their large pore sizes and strong acidity, which facilitate macromolecular cracking into shorter hydrocarbons.^157,156^ Ni-based catalysts further enhance local microwave absorption, improving heating efficiency, while activated carbon provides high surface area and uniform heat distribution, promoting selective reactions and minimizing coke formation.^158^

Catalyst properties including acidity, porosity, and chemical composition significantly influence product yield and selectivity. Acidic zeolites and metal-doped catalysts generally increase oil yields,^159^ whereas basic and Ni-based catalysts promote gas formation.^61,160^ As shown in Tables S7 and S8, different catalysts applied to the same plastic type produce varied product distributions due to these intrinsic differences,^100,161^ as well as the influence of process temperature,^162^ and plastic type.^163^

The structural nature of plastic also affects catalytic behavior. LDPE and HDPE, despite being chemically similar, differ in branching, crystallinity, and dielectric response, all of which alter microwave energy absorption and degradation kinetics. Branched polymers degrade at lower temperatures and exhibit faster decomposition, yielding a broader spectrum of volatile hydrocarbons likeolefins, aromatics, and short-chain alkanes.^13^ In contrast, linear HDPE produces more saturated hydrocarbons such as linear alkanes and 1-alkenes.^164^ Fan et al.,^165^ observed that PP, a branched plastic, produced diverse aliphatic hydrocarbons (C_8_–C_16_), while HDPE yielded simpler saturated fractions, highlighting how plastic structure dictates product composition.

Catalytic microwave pyrolysis experiments typically operate between 300 and 700 °C. For LDPE, bentonite achieved the highest oil yield (87.6%),^62^ whereas other catalysts resulted in lower yields. A substantial solid yield (71.5%) was observed with HDPE and Al_2_O_3_–ZnFe_2_O_4_.^166^ A notable example is Ru/ZSM-5 (4 wt % Ru), which delivered 88 wt % gas yield under microwave heating (900 W, 300 °C, 30 min), maintaining stability over seven cycles—far superior to conventional pyrolysis, which saw a drop from 72 wt % to 45 wt % gas after one cycle.^167^ Microwave heating also enhanced olefin selectivity, producing BTX-rich liquids dominated by benzene, toluene, and xylenes according to their report.^167^

Shen et al.^168^ investigated Fe-based catalysts for HDPE pyrolysis and found that FeAlOx (22%) achieved the highest gas yield (94%), with >98% H_2_ composition, followed by FeAlOx (7%) and Fe/AC. The strong C–H bond dissociation ability of FeAlOx was attributed to synergistic Fe–Al–O interactions that promoted hydrogen abstraction and hydrocarbon chain propagation. The 22% Fe loading provided optimal active site density, moderate acidity, and effective hydrogenation, yielding long-chain alkanes while minimizing cracking. Additionally, Fe-based catalysts produced CNTs with diameters ranging from 26–141 nm, depending on the support, showing that catalyst composition also influences CNT morphology.

Luo et al. investigated Fe, Ni, and Fe–Ni catalysts supported on SiC (Fe/SiC, Ni/SiC, Fe_1_–Ni_1_/SiC, Fe_2_–Ni_1_/SiC, Fe_1_–Ni_2_/SiC) for LDPE microwave pyrolysis under 1800 W, 800 °C, and a 1:2 catalyst-to-plastic ratio. Fe-containing catalysts, particularly Fe–Ni/SiC (2:1), achieved the highest gas yield (73.61 wt %), a 44.65 wt % improvement over single-metal systems. The enhanced activity was attributed to Fe–Ni synergism that promoted catalytic cracking and dehydrogenation, yielding 73.89 vol % H_2_—154% higher than non-catalytic pyrolysis. Oil composition showed that bimetallic catalysts favored smaller hydrocarbons due to stronger C–C bond cleavage, whereas single-metal systems exhibited milder cracking. The Fe–Ni/SiC (2:1) catalyst produced the highest gasoline-range fraction (52.44%) through deep cracking and aromatization, while Fe–Ni/SiC (1:1) yielded the most diesel-range products (38.86%) owing to moderate cracking. Among single metals, Fe/SiC produced 31.56% gasoline and 31.91% diesel, while Ni/SiC gave 29.57% gasoline and 35.88% diesel, consistent with Ni’s hydrogenation selectivity.^169^

Peng et al. examined LDPE conversion to liquid fuels using MCM-41, HY zeolite, and a composite MCM-41/HY (2:1) catalyst at 450 °C. HY produced the highest gas yield (59.00 wt %) due to its strong Brønsted acidity and small pores (∼0.74 nm), which enhanced β-scission and secondary cracking. In contrast, MCM-41 gave the highest oil yield (61.25 wt %) and gasoline-range fraction (C_5_–C_12_: 92.63%) owing to its meso-porosity (2–10 nm) and moderate acidity, enabling controlled cracking and aliphatic formation (mainly C_6_–C_7_ alkanes). The MCM-41/HY (2:1) catalyst achieved a balance, yielding 63.75 wt % oil and higher monoaromatic selectivity due to combined meso-porosity and acidity, which promoted aromatization without excessive cracking. Accordingly, HY-derived oil was rich in aromatics (toluene, xylene), while MCM-41 produced mainly linear aliphatic because its weaker acidity limited cyclization and aromatic formation.^170^

Shoukat et al. used magnetic catalysts (Al_2_O_3_–NiFe_2_O_4_, Al_2_O_3_–ZnFe_2_O_4_, Al_2_O_3_–MgFe_2_O_4_) for HDPE microwave pyrolysis into CNTs and hydrogen. Al_2_O_3_–NiFe_2_O_4_ produced the highest CNT yield (78 wt %), surpassing Al_2_O_3_–ZnFe_2_O_4_ (71.5 wt %) and Al_2_O_3_–MgFe_2_O_4_ (74 wt %), due to Ni’s strong graphitization activity. Al_2_O_3_–ZnFe_2_O_4_ generated the highest H_2_ content (90 vol %) and gas yield (19.5 wt %), reflecting its high oxygen mobility and redox capacity that favored gas-phase reforming. Its moderate acidity and weak hydrocarbon adsorption suppressed oil formation, while yielding heavier oil fractions by limiting secondary cracking. The overall liquid composition across catalysts was dominated by C_5_–C_22_ hydrocarbons, indicating selective midrange cracking under microwave conditions and balanced catalytic activity without excessive gasification.^166^

Yao et al. studied hydrogen and syngas generation from HDPE using zeolite-based and Ni-loaded catalysts (ZSM-5–30, β-zeolite-25, Ni/ZSM-5–30, Ni/β-zeolite-25, Ni/Y-zeolite-30) in a two-stage microwave reactor. Ni/ZSM-5–30 achieved the highest H_2_ yield (66.09 mmol g^–1^ plastic), attributed to the combined effects of Ni’s reforming activity and ZSM-5′s strong acidity and shape selectivity, which enhanced polymer cracking and water–gas shift reactions. In contrast, β-zeolite-25 produced the lowest H_2_ yield (53.87 mmol g^–1^) due to its weaker acidity and less favorable pore structure. The syngas yield followed the order Ni/ZSM5–30 > Ni/β-zeolite-25 > Ni/Y-zeolite-30 > ZSM5–30 > β-zeolite-25, consistent with variations in Ni loading, acidity, and pore accessibility influencing reforming efficiency.^171^

Collectively, these studies demonstrate that catalyst composition, acidity, and pore architecture critically influence product yield and distribution in MAPP. Fe–Ni bimetallic systems and Ni-based zeolites enhance hydrogen generation and light hydrocarbon formation, while mesoporous silicas (e.g., MCM-41) and hybrid structures balance cracking and aromatization. Despite these advances, cross-study comparison is limited by differences in feedstock, particle size, catalyst loading, and pyrolysis parameters. Future studies should adopt standardized conditions including consistent plastic type, particle size, catalyst-to-plastic ratio, and microwave parameters to isolate catalyst effects. Systematic evaluation of acidic (zeolite, silica), basic (alkaline oxides), and amphoteric (FeAlO_*x*_) catalysts under identical settings would enable direct comparison of gas, liquid, and char yields and elucidate how catalyst acidity or basicity governs hydrocarbon distribution in the liquid fraction. Such harmonized methodologies are essential for establishing reliable structure–activity–performance relationships and advancing the design of efficient catalysts for MAPP.

#### 4.2.3 Effect of Pyrolysis Method

MAPP employs microwave susceptors to overcome plastics’ weak microwave absorption. Two main methods—pre-mixed and non-premixed—are used, each producing distinct heating patterns and influencing product yields and composition.

In the pre-mixed method, both plastic and susceptor absorb microwave energy, leading to rapid heating and higher reaction temperatures. Zhang et al.^128^ described it as a “flash” process that enhances thermal uniformity and conversion efficiency, while the non-premixed method relies solely on heat transfer from the susceptor to the plastic, resulting in slower and less uniform heating and different product profiles. Undri et al.^13^ applied the pre-mixed approach to PS using carbon powder as a susceptor at 3.0–6.0 kW, yielding 86.5 wt % liquid rich in single-ring aromatics (C_6_–C_10_: 93.9%, styrene: 66.0%) and 3.0 wt % gas suitable for energy recovery. In contrast, Hussain et al.^172^ used a non-premixed setup with 700 W power and copper wire gauze, obtaining 80% liquid and 15% gas from PS, mainly containing styrene, ethylbenzene, and polyaromatic compounds.

Suriapparao and Vinu^123^ studied LDPE, LLDPE, and PP with various susceptors (graphite, activated carbon, aluminum, fly ash, SiC) at polymer/susceptor = 100:1 and 100–800 W microwave power. At 450 °C with graphite, light hydrocarbons (C_3_–C_6_) reached 7.4–20.99 wt % (PP), 0–20.63 wt % (LDPE), and 0–50.26 wt % (LLDPE), while total gas yields ranged from 50–60 wt %. Yang and Ani^173^ reported that tire crumb pyrolysis with activated carbon produced 54.39 wt % oil at 500 °C (42.59 MJ/kg), suitable as a petroleum substitute. Undri et al.^13^ found HDPE yielded more liquid (80.2 wt %) than PP (56.2 wt %), with HDPE oil rich in linear alkanes and PP oil containing branched alkanes and aromatics.

In the non-premixed method, Wan Mahari et al.^174^ copyrolyzed HDPE with used frying oil (UFO) over activated carbon, achieving 81 wt % liquid at 500 °C and 800 W, mainly aliphatic (C_8_–C_24_) with 42–46 MJ/kg energy content. Similarly, Zhao et al.^175^ utilized the nonpremixed method for copyrolysis of PP and moso bamboo sawdust over an HZSM-5 catalyst. At 250 °C, a bamboo/PP ratio of 1:2, and a feedstock/catalyst ratio of 2:1, a maximum bio-oil yield of 61.62 wt % was achieved, containing aliphatic hydrocarbons, aromatics, and oxygenates.

## 5 Applications of Products from Microwave Plastic Pyrolysis

MAPP produces solid, liquid, and gaseous fractions, each with distinct applications. Although MAPP has yet to reach industrial implementation and requires further development,^176^ laboratory-scale studies reveal that these products possess promising fuel and material characteristics.

### 5.1 Liquid Fraction

The liquid fraction from MAPP consists primarily of hydrocarbons,^177^ including alkanes, alkenes, alkynes, and aromatics (e.g., benzene, toluene), resembling conventional fossil-derived fuels.^170^ Table S9 presents the physical properties of oil from MAPP, such as viscosity, density, heating values, and American Petroleum Institute (API) gravity. These properties align closely with the acceptable ranges for commercial diesel (BS IV 2017 Nationwide) and gasoline (BS IV 2017 Nationwide): kinematic viscosity (2.0–4.5 × 10^–6^ m^2^/s), density (0.78–0.84 g/cm^3^), calorific heating value (43–46 MJ/kg), and API gravity (30–40).^121^ Fan et al.^165^ recovered pyrolysis oil with aviation fuel characteristics through microwave pyrolysis of mixed plastics over HZSM-5. Similarly, LDPE pyrolyzed over MgO and hydrotreated with Ni/biochar yielded fuel with freezing (−62 °C) and flash points (59 °C) comparable to jet fuel standards (−47 °C; 38 °C).^178^ The diverse functional groups within this oil fraction (Table S4) make it a valuable precursor for dyes, resins, coatings, adhesives, and disinfectants.

### 5.2 Gas Fraction

The gas fraction typically the second-largest product (Tables S4–S6) and contains light hydrocarbons (C_1_–C_3_), CO, CO_2_, and H_2_. Multiple studies have emphasized hydrogen generation as a key advantage of microwave pyrolysis.^15,179^ Hydrogen serves as a clean fuel and feedstock for ammonia and methanol synthesis, though purification remains costly. Impurities (CO, CO_2_) necessitate upgrading via pressure swing adsorption, membrane separation, or cryogenic distillation. Other gaseous products such as methane, ethane, and propane are valuable as fuels for heating, cooking, and power generation. Additionally, syngas (CO + H_2_) recovered from MAPP can be directly used for electricity production or as a feedstock for Fischer–Tropsch synthesis.^69^

### 5.3 Solid Fraction

The solid fraction, mainly carbonaceous char,^133^ is porous due to the rapid, volumetric heating characteristic of microwaves, which enhances pore formation.^180^ For instance, char from PET and rice husk copyrolysis exhibited a surface area of 197 m^2^/g and a pore volume of 0.08 cm^3^/g^124^ prior activation. This char holds potential as an adsorbent or precursor for pollutant removal. Char from PE–food waste pyrolysis adsorbed 0.27 mg/g of metformin, rising to 0.34 mg of metformin per gram after activation with K_3_PO_4_.^181^ Microwave-assisted PET depolymerization char achieved 213 mg/g Cu (II) adsorption at neutral pH, surpassing most Cu adsorbents.^124^ Similarly, Fe-modified PET char produced using FeCl_2_·4H_2_O and NaBH_4_ reached a tetracycline adsorption capacity of 105.26 mg/g.^182^ However, studies exploring microwave plastic char for adsorbing dyes, VOCs, antibiotics, and trace metals remain limited. Another valuable carbon product is CNTs, widely obtained from catalytic MAPP.

Shen et al.^168^ synthesized CNTs with diameters of 53–76 nm from HDPE using FeAlOx catalysts, with their formation attributed to localized microwave “hotspots.” CNTs with diameters in the range of 25–93 nm was also produced from HDPE, PP, and their mixtures using ZSM-5 catalysts.^183^ Fe–Ni/Al_2_O_3_ catalysts yielded 445 mg CNTs g^–1^ LDPE, which increased to 513 mg g^–1^ after ten cycles, with an average CNT diameter of 25 nm.^184^ CNT yields vary with catalyst type—Fe/Al_2_O_3_, Ni/Al_2_O_3_, Co/Al_2_O_3_, and FeCo/Al_2_O_3_ produce different morphologies and carbon recoveries. LDPE pyrolysis with FeNi/Al_2_O_3_ achieved 445 mg g^–1^,^184^ HDPE + Fe/FeAl_2_O_4_ produced 120 mg g^–1^,^185^ while up to 5.8 and 5.0 g g^–1^ catalyst were reported for LDPE and PP, respectively.^186^ NiFe_2_O_4_ and FeAlOx catalysts yielded significantly higher outputs—1560 mg g^–1^ catalyst and 1560 mg C g^–1^ plastic, respectively.^166,15^ The formation of CNTs during catalytic MAPP involves a series of processes, including thermal decomposition, catalytic cracking, and carbon rearrangement, as detailed in another review.^187^

### 5.4 Future Opportunities for Generating High-Value Products from Plastics

Recent studies on MAPP highlight its potential to transform plastic waste into valuable gas, liquid, and solid products. These include hydrocarbon-rich oils for fuels and chemical feedstocks, high-purity hydrogen for energy and industrial use, and carbon-based materials such as activated char and carbon nanotubes with adsorption and catalytic applications.^188,168^ However, achieving large-scale implementation requires overcoming key technical, economic, and sustainability barriers. Future progress depends on advances in catalyst development, reactor design, material optimization, and process integration and optimization. As summarized in Table 1, these innovations are essential for realizing MAPP’ role in a circular plastics economy, as previously proposed by Jehanno and coauthors.^189^

**Table 1:** Future Opportunity for Transforming Plastic Waste to Value-Added Products and Their Significance

Future opportunity	Significance
Develop selective and scalable reactions for real-world plastic waste	Enables the transformation of chemically stable plastics into valuable products despite impurities; critical for industrial-scale upcycling.
Design efficient, selective, and robust catalysts/reagents	Reduces energy consumption and increases compatibility with mixed and contaminated plastic streams, making upcycling more practical and sustainable.
Shift research from virgin polymers to real plastic waste	Increases the real-world applicability of academic innovations by accounting for additives and contaminants in postconsumer plastics.
Integrate LCA, TEA, and material flow analysis (MFA) early in research	Ensures that economic viability, environmental impact, and market fit of upcycled products are considered from the start, guiding meaningful innovation.
Focus on low-volume, high-value upcycled products	Provides economic justification even for small-scale upcycling, which can support cost-intensive recycling processes and promote broader recycling rates.
Use sustainability metrics to justify use of plastic waste over virgin feedstocks	Empowers data-driven decision-making to prioritize waste-derived feedstocks that provide net environmental and economic benefits.
Design upcycled materials with built-in recyclability	Facilitates further recycling at end-of-life, moving toward a closed-loop system and extending material lifetimes.
Create polymers with degradable monomers or dynamic bonds	Enables controlled depolymerization and repolymerization, giving rise to customizable and fully recyclable high-performance materials.
Demonstrate closed-loop upcycling in thermosets and copolymers	Produces recyclable materials with differentiated properties from mixed or single-use waste streams, expanding the scope of circular plastics.
Innovate in upcycling to produce specialty polymers, functional molecules, and performance materials	Allows for high-value outputs that exceed the quality of commodity plastics, unlocking new commercial and technological opportunities.
Combine upcycling with a holistic plastics management strategy	Positions upcycling as one part of an integrated solution to the plastic waste crisis, supporting broader sustainability and circular economy goals.

## 6 Life Cycle Analysis of Microwave Plastic Pyrolysis into Value-Added Product

MAPP presents a promising approach for plastic waste valorization, offering both waste reduction and resource recovery benefits. By utilizing abundant and inexpensive feedstocks, this process can deliver significant economic and environmental advantages. However, large-scale implementation requires comprehensive LCA to evaluate its environmental performance, considering inputs such as feedstock, energy, catalysts, and outputs including gases, liquids, and solids, some of which may pose secondary waste concerns. Both conventional and microwave pyrolysis are thermochemical recycling methods but differ in operating conditions—temperature, heating mechanism, energy input, and product distribution—which strongly influence LCA outcomes. Conventional catalytic pyrolysis typically operates at 500–800 °C, relying on external heating for uniform temperature distribution. In contrast, catalytic microwave pyrolysis generally occurs at 300–500 °C, where microwave energy directly heats polar materials and catalysts, improving thermal efficiency and reducing energy demand.^190^

Pressure conditions are similar for both processes, typically near atmospheric levels. However, microwave pyrolysis can generate localized hot spots, leading to temporary pressure variations that accelerate reaction kinetics.^191^ Residence time is another differentiator: conventional catalytic pyrolysis usually requires 30–60 min, whereas microwave-assisted processes complete within 5–15 min due to selective and volumetric heating. Moreover, microwave pyrolysis consumes 30–50% less energy, as heat is generated internally and selectively, minimizing thermal losses and improving energy efficiency per unit of plastic processed.

Although limited, existing LCAs of microwave pyrolysis—mainly on copyrolysis with biomass—have shown encouraging results in terms of emission reductions and energy recovery. Co-pyrolysis improves product quality, increases oil and syngas yields, and reduces emissions since biomass is often considered carbon neutral.^192,193^

Muniyappan et al.^194^ conducted an LCA on microwave copyrolysis of mixed biomass and waste electrical and electronic plastics (WEEP). Optimized through response surface methodology (RSM) and artificial neural network (ANN) modeling, the study reported emissions of 771.95 kg CO_2_ equiv, 3.11 kg SO_2_ equiv, 1.69 kg PO_4_ equiv, and 338.59 dichlorobenzene equiv per tonne of feedstock. The microwave reactor and drying units were responsible for 97.64% of total GHG emissions. Nonetheless, overall CO_2_ emissions were 54.7% lower than conventional pyrolysis of municipal solid waste (1,194 kg CO_2_ equiv),^195^ and markedly below those of incineration (6,639.8 kg CO_2_ equiv), anaerobic digestion (3,245 kg CO_2_ equiv), or landfilling (5,475.9 kg CO_2_ equiv).

Similarly, Lee et al.^196^ assessed the LCA of microwave steam pyrolysis for recovering ethylene and propylene from PE and PP. Their findings showed GWP100 and GTP100 values of 2.55 and 2.46 kg CO_2_ equiv/kg C_2_H_4_, respectively—a 90% reduction compared to conventional processes. Neha et al.^197^ examined microwave copyrolysis of food waste and LDPE, which yielded 42 wt % bio-oil, 42 wt % biochar, and 16 wt % gas. The LCA revealed emissions of 38.92 kg CO_2_ equiv and 0.048 kg SO_2_ equiv per 100 kg feedstock, with the drying and pyrolysis stages contributing 91% of energy consumption and environmental impact.

A distinctive feature of MAPP is the high feed-to-catalyst ratio, necessary due to plastics’ poor microwave absorption. Heat generation mainly depends on microwave-absorbing materials (catalysts or susceptors such as SiC), which transfer heat to the plastic via conduction. Maintaining a high feed: catalyst ratio prevents localized overheating, ensuring uniform heating and efficient thermal conversion.^198^ Conversely, excessive catalyst loading can promote undesired polymerization and coke formation, reduce product quality and shorten catalyst life. Hence, an optimized ratio enhances selectivity for valuable liquids while improving process sustainability.^199^

Monteiro et al.^200^ performed a comparative LCA on catalytic pyrolysis of HDPE and high-impact polystyrene (HIPS) using H-USY and SO_4_^2–^/SnO_2_ catalysts. While catalysis improved product quality, it did not always yield environmental benefits. Reported GWP ranged from 1.31–3.34 kg CO_2_ equiv/kg plastic, acidification potential from 4.0–8.1 g SO_2_ equiv, eutrophication from 0.5–1.5 g PO_4_^3–^ equiv, and ozone depletion potential of 1.6 mg CFC-11 equiv. Notably, human toxicity impacts were substantial. Increasing catalyst loading significantly raised environmental burden as GWP rose by over 50% under high catalyst scenarios, highlighting the importance of feed: catalyst optimization

A related LCA on microwave-assisted slurry pyrolysis using solid acid catalysts achieved ∼ 88% olefin yields under mild conditions (350–375 °C). Environmental indicators improved markedly: GWP decreased by 817.6 kg CO_2_ equiv per functional unit relative to conventional systems, with acidification, eutrophication, and ozone depletion potentials reduced by 65, 47, and 70%, respectively. Human and ecotoxicity impacts also declined, primarily due to lower energy use and reduced catalyst demand. These improvements were linked to the high feed: catalyst ratio and reactor design, which enhanced heat transfer and minimized catalyst regeneration, making the process both environmentally and economically favorable.^201^

Dai et al.^202^ further evaluated a catalytic microwave-assisted pyrolysis system producing naphtha. Their comprehensive LCA confirmed substantial GWP reductions (817.6 kg CO_2_ equiv per functional unit) and overall improvements in acidification, eutrophication, and ozone depletion impacts, though detailed category values were not reported. The study attributed these benefits to optimized operating parameters, particularly high feed: catalyst ratios, which extended catalyst lifetime, reduced regeneration frequency, and minimized embodied energy and emissions. The authors emphasized that process stability across multiple cycles also lowered the cumulative environmental burden.^99^

While current LCAs on microwave copyrolysis of plastics and biomass demonstrate promising environmental credentials, these results cannot be directly extrapolated to pure plastic pyrolysis due to differing feedstock characteristics. Biomass cofeeds alter system performance—affecting heating efficiency, gas composition, and emission profiles—and therefore influence LCA outcomes. Consequently, there is an urgent need for dedicated LCAs focusing solely on microwave pyrolysis of pure plastics to enable accurate benchmarking against conventional recycling and disposal routes.

Such studies should account for how product distribution affects environmental outcomes. Higher gas yields enhance energy recovery but may increase emissions if not captured effectively. Pyrolysis oil can displace fossil fuels but may require further refining, adding environmental burden. Char offers potential for carbon sequestration if applied to soils or cement composites but can pose disposal issues if landfilled. These trade-offs depend on process parameters: lower temperatures and shorter residence times favor liquids and solids, whereas higher temperatures and extended durations increase gaseous yields.^152^

Comprehensive LCAs will enable the identification of optimal operating conditions and system designs to minimize environmental impacts. This includes integrating renewable or low-carbon electricity to power microwave systems, thereby reducing process emissions,^203^ and combining pyrolysis with carbon capture and utilization technologies.^204^ Additionally, LCAs should encompass upstream and downstream activities such as feedstock sorting, cleaning, product purification, and byproduct management, which often contribute significantly to overall impacts.

## 7 Comparison of Microwave and Conventional Plastic Pyrolysis

Microwave-assisted pyrolysis offers several advantages over conventional pyrolysis, including lower operating temperatures (≤500 °C), faster heating rates (up to 50 °C/min), shorter reaction times (typically <10 min), and reduced energy consumption.^18^ Although higher temperatures can be achieved, identifying effective susceptors remains a challenge.

Prathiba et al.^121^ compared the pyrolysis of waste PS using activated carbon under both methods. Microwave pyrolysis at 450 W (PS: activated carbon = 10:1) yielded 93.04 wt % oil, 1.22 wt % char, and 5.74 wt % gas, outperforming conventional pyrolysis (83.44 wt % oil, 5.94 wt % char, 11.08 wt % gas). The oil from microwave heating exhibited lower viscosity (1.67 × 10^–6^ m^2^/s) and higher aromatic and alkene content (50.98 wt % and 8.44 wt %, respectively), enhancing its suitability as a fuel precursor.

Selvam et al.^201^ reported the conversion of LDPE using a SiC monolith catalyst, achieving 96% conversion under microwave conditions compared to 46% conventionally. Coke deposition was ∼ 49% lower in the microwave system due to suppressed hydrogen transfer reactions, and the oil product contained >75% medium-chain olefins versus 30% under conventional heating. Similar benefits were observed for HDPE pyrolysis over SiC, where microwave heating improved conversion efficiency and product quality.^139^

Wang et al.^205^ examined polyolefin-rich plastic waste using HZSM-5 under both heating modes. Microwave pyrolysis produced ∼ 78 wt % liquid yield (vs ∼ 65 wt % conventional) with higher selectivity toward light olefins (C_5_–C_11_) and monoaromatics, whereas conventional heating favored heavy hydrocarbons and alkanes. The superior performance of MAP was attributed to rapid, uniform volumetric heating, which minimized secondary cracking and coke formation while improving energy efficiency and catalyst recyclability.

Zhao et al.^206^ developed a Fe-based magnetically susceptible catalyst designed for efficient microwave absorption. Compared to conventional pyrolysis, microwave operation produced >60% BTX in the liquid phase versus <30% under conventional heating. Tar, wax, and coke formation were also ∼ 50% lower. These improvements were linked to nonthermal microwave effects—localized superheating and bond polarization—that enhanced selective C–C and C–H bond cleavage while preventing polymerization. The process also showed higher energy efficiency due to rapid, uniform heating by the Fe-based catalyst.

Figure 5 (a) and (b) illustrates distinct gas and oil yield trends for different plastics and catalysts under both heating methods. HDPE pyrolysis over ZSM-5 under conventional heating produced 83.15 wt % oil,^207^ compared to only 12 wt % under microwave heating.^208^ Conversely, much higher oil yields were achieved during microwave pyrolysis of PS (93.04 wt %), HDPE/vegetable oil blends (84 wt %), and mixed plastics (63.75 wt %) over activated carbon and NiO.^121,209,170^

**Figure 5:**
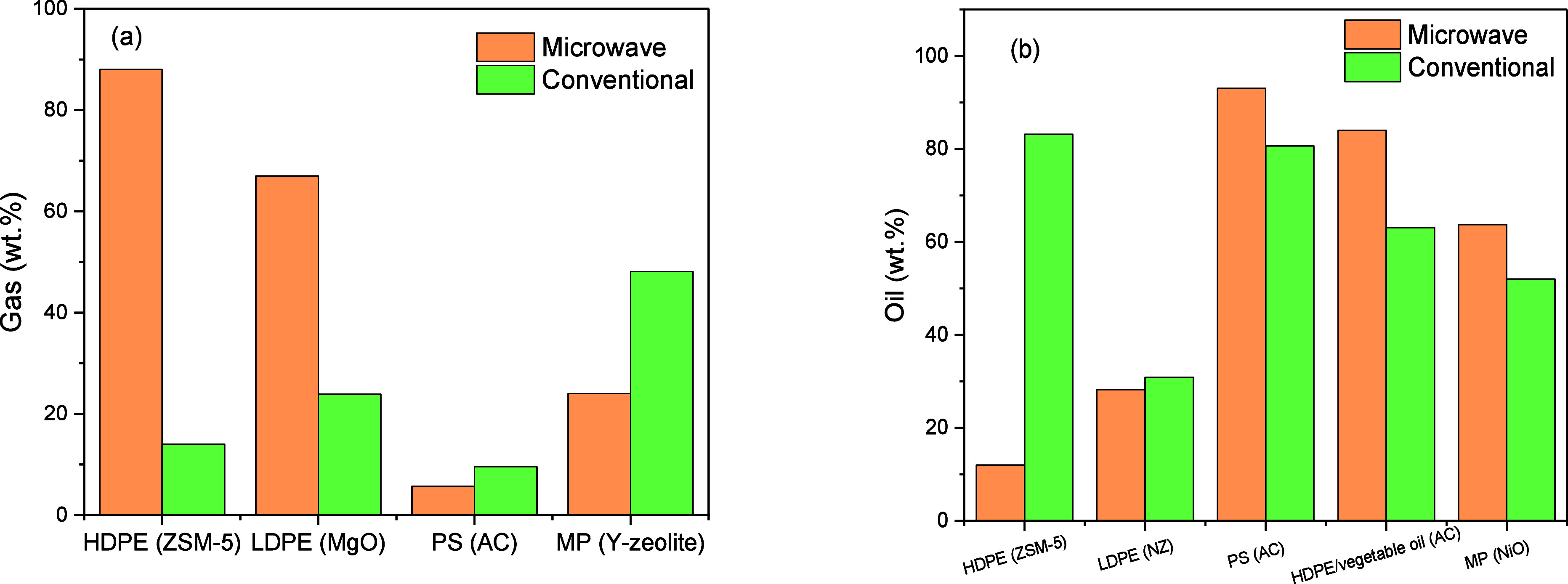
(a) Gas and (b) oil yields from plastic pyrolysis under conventional and microwave heating (NZ-natural zeolite; MP-mixed plastic; AC-activated carbon).

Gas production also varied with heating mode and catalyst type. Microwave pyrolysis of LDPE, HDPE, and PS using ZSM-5, MgO, or activated carbon generally yielded more gas than conventional methods, due to enhanced primary and secondary cracking. However, in mixed plastic pyrolysis over Y-zeolite, conventional heating produced 48.1 wt % gas^100^ versus 24 wt % under microwave conditions.^170^ These variations highlight the influence of heating mechanisms on product distribution.^100^

Catalyst stability consistently emerges as a key differentiator. Microwave systems typically maintain higher long-term catalytic performance due to uniform volumetric heating that minimizes coke formation—a major cause of deactivation. Zhao et al.^206^ demonstrated that their Fe-based catalyst retained 91.7% activity after five microwave cycles versus 68.3% under conventional heating, with 56.7% less coke deposition. Similarly, Wang et al.^205^ reported 72.6% selectivity toward light olefins and aromatics under microwave heating compared to 58.9% conventionally. After three regenerations, microwave-treated catalysts showed less structural degradation and 42.3% lower coke accumulation.

Selvam et al.^201^ observed that LDPE pyrolysis under microwave heating maintained >90% catalyst activity over four cycles, compared to a 35% decline in the conventional system. Heavy coke deposition was 49.1% lower due to reduced hydrogen transfer and more selective cracking enabled by rapid, even heating. Although Praithiaba et al.^121^ did not assess catalyst degradation directly, their findings; higher oil yield and lower residue, suggest enhanced operational stability under microwave conditions.

Figure 6 illustrates oil yield variations in LDPE pyrolysis using different catalysts under both heating methods. The results underscore the catalyst-specific response to microwave irradiation. For instance, MgO achieved higher oil yield under conventional (72 wt %)^210^ than under microwave (30.3 wt %),^137^ whereas activated carbon and NiO favored microwave heating, yielding 82.16 wt %^211^ and 56.53 wt %,^129^ respectively, compared to 73.1 wt %^212^ and 23.61 wt %^213^ conventionally. Natural zeolite exhibited similar yields in both heating methods.^135,214^ These findings suggest that while microwave heating can enhance catalytic efficiency in certain cases, it may also alter vapor-phase reactions, leading to variations in oil yields compared to conventional pyrolysis.

**Figure 6:**
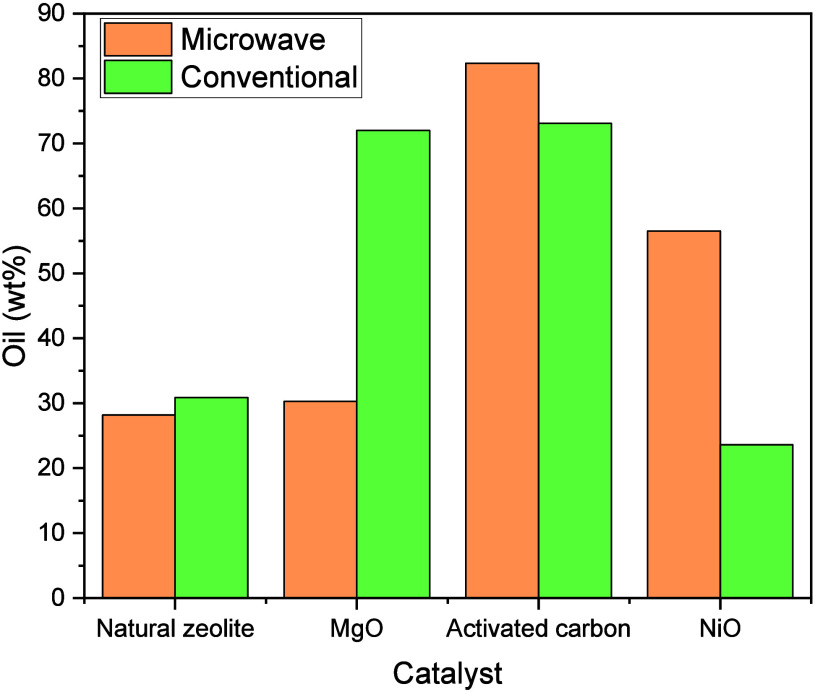
Pyrolysis of LDPE over different catalysts under conventional and microwave heating.

### 7.1 Comparing Product Composition between Conventional and Microwave Plastic Pyrolysis

Microwave and conventional pyrolysis of plastics yield oil products with distinct compositions, influenced by heating mechanisms, catalysts, and reactor design. Juliastuti et al.,^135^ processed LDPE from multilayer packaging waste via microwave pyrolysis using natural zeolite, achieving 19% hydrocarbon yield at 550 °C after 90 min. A long residence time and higher temperatures promoted hydrocarbon enrichment. Khaghanikavkani et al.^139^ employed a rotating microwave reactor with silicon carbide absorber for mixed plastics, but the liquid produced was unsuitable for phase-change materials, prompting the need for additional fractionation.

Lam et al.^209^ copyrolyzed HDPE with used cooking oil under microwave vacuum conditions (581 °C, 20 min) using activated carbon. The oil was rich in light hydrocarbons, low in heteroatoms, and exhibited a high energy value (49 MJ/kg), exceeding that of diesel and gasoline. Similarly, Peng et al.^170^ performed continuous microwave pyrolysis of LDPE over a dual-bed MCM-41/HY zeolite system (2:1) at 450 °C, yielding 78.21 wt % monocyclic aromatic hydrocarbons (MAHs). Although zeolite catalysts may reduce liquid yield, they enhance oil quality and calorific value, demonstrating the importance of acidity and pore structure in producing high-value fuel fractions.

For PS, Prathiba et al.^121^ compared microwave (330 °C, 5.5 min, 450 W) and conventional pyrolysis (418 °C, 60 min) with activated carbon. Microwave treatment produced a highly aromatic liquid containing 8.44% alkenes, 0.96% α-methyl styrene, 23.21% condensed aromatics, and 26.77% benzene derivatives, with a calorific value of 45 MJ/kg, higher than that of the conventional process (39 MJ/kg).

Li et al.^215^ used Ni-supported catalysts on Al_2_O_3_, TiO_2_, ZrO_2_, and Nb_2_O_5_ in a two-stage fixed-bed reactor to pyrolyze polyethylene, focusing on carbon nanomaterial formation. Ni/Al_2_O_3_ produced up to 36.5 wt % carbon nanotubes. In contrast, Onwudili et al.^100^ examined conventional catalytic pyrolysis of mixed plastics with Y-zeolite and ZSM-5 (500–600 °C). Although catalytic runs yielded less oil than noncatalytic ones, Y-zeolite produced >90% aromatic hydrocarbons (C_5_–C_15_), suitable for transport fuels. Hydrogen recovery varied with catalyst and temperature: Y-zeolite (ZY-1, Si/Al = 5.2) yielded the most H_2_ (22.2–31.8%), while ZSM-5 and FCC produced lower H_2_ but higher C_2_–C_4_ hydrocarbons. These results underscore the importance of acidity, Si/Al ratio, and surface area in directing selectivity toward dehydrogenation or cracking pathways.

Ratnasari et al.^207^ used a dual-catalyst system (MCM-41/ZSM-5, 1:1) in conventional two-stage pyrolysis of HDPE, producing 95.85 wt % aromatic oil dominated by C_8_–C_12_ gasoline-range hydrocarbons (97.72%). The gas fraction contained mainly C_2_–C_4_ olefins, demonstrating the efficiency of meso–microporous catalysts for fuel-range products. In contrast, Selvam et al.^201^ developed a microwave-assisted slurry pyrolysis of LDPE at 350–375 °C using solid acid catalysts (Pt-HY, P-SiO_2_, Al-MCM-41) supported on a SiC monolith. The process achieved ∼ 88% olefins (C_4_–C_12_) in the liquid phase, with low coke formation and light gas (C_2_–C_4_) generation, indicating improved thermal management under microwave conditions.

Fan et al.^137^ performed microwave pyrolysis of LDPE using activated carbon without a catalyst. The liquid contained C_5_–C_20_ alkanes, alkenes, and aromatics, while gases comprised light hydrocarbons and permanent gases (H_2_, CO). The char residue suggested incomplete conversion or localized overheating. Similarly, Huo et al.^210^ combined NiO and ZSM-5 under microwave heating for HDPE pyrolysis, producing aromatic-rich oil (C_5_–C_20_) and light alkanes/alkenes (C_1_–C_4_), highlighting efficient catalytic cracking and aromatization.

Jing et al.^211^ optimized heat distribution through intermittent microwave heating of HDPE using activated carbon and molecular sieves (MS4A, MS13X). The products included C_7_–C_20_ waxes and oils, gaseous C_1_–C_6_ hydrocarbons, and carbonaceous char. Qie et al. (233) employed hierarchically structured ZSM-5 modified by microwave chelation for HDPE pyrolysis, achieving higher alkane-to-olefin ratios and stable catalyst recyclability, with gas products dominated by ethylene.

Budsaereechai et al.^62^ conducted catalytic pyrolysis of PS, PP, LDPE, and HDPE in a fixed-bed reactor with bentonite clay at 500 °C. PS yielded ∼ 95% aromatic oil resembling gasohol-91, while polyolefins produced diesel-like aliphatic hydrocarbons with high energy content (∼44 MJ/kg). PS-derived oil exhibited favorable engine performance and emissions. Paucar-Sánchez et al.^216^ used postconsumer plastic waste with Sepiolite and Montmorillonite catalysts at 500 °C, producing paraffin- and aromatic-rich oils like diesel/kerosene fuels. Catalyst acidity influenced selectivity, with gaseous products dominated by CO_2_, CO, CH_4_, and light hydrocarbons.

Microwave pyrolysis of HDPE over ZSM-5 generates aromatic- and BTX-rich oil,^208^ while conventional pyrolysis with the same catalyst yields predominantly aromatic hydrocarbons (96 wt %).^207^ Similarly, microwave pyrolysis of LDPE over MgO generates oil containing monoaromatics and alkenes,^137^ while conventional pyrolysis produces paraffin- and naphthalene-rich oil.^210^ LDPE pyrolysis with activated carbon under microwave heating results in C_7_–C_20_ hydrocarbons, whereas conventional methods favor alkanes over aromatics.^211,212^ For PS, microwave pyrolysis enhances aromatic content (C_9_–C_24_), while conventional methods favor olefins (C_8_–C_11_).^121^ Nonetheless, mixed plastic pyrolysis over zeolite shows comparable gasoline-range hydrocarbon yields under both heating modes.^170,100^

The comparison of yields and composition of oil from catalytic microwave and conventional pyrolysis of plastics highlighted is based on findings from different studies, often conducted under varied experimental conditions. As a result, these comparisons may not provide a fully accurate assessment of the differences between the two pyrolysis methods. Notably, direct comparative studies that systematically evaluate product yields and oil composition under identical experimental conditions remain scarce in the literature. Therefore, further research is needed to establish a more definitive understanding of how heating methods influence pyrolysis product yields and composition.

## 8 Challenges of Microwave Pyrolysis of Plastic

Microwave-assisted pyrolysis of plastics faces significant technical challenges. Most plastics are microwave-transparent, making direct heating inefficient and yielding minimal temperature rise (<5 °C) even at maximum power.^217^ To achieve decomposition temperatures, microwave susceptors are introduced for hybrid heating; however, this often generates hotspots that reduce efficiency,^218^ cause uneven heating, thermal runaway, safety risks,^127^ and potential equipment damage.^18^ Such nonuniform temperature distribution complicates measurement,^18^ leading to inconsistent product composition and poor reproducibility in MAPP.

Effective hybrid heating depends on heat transfer from the irradiated susceptor to the plastic, governed by the susceptor’s dielectric properties and microwave penetration depth.^219^ Variations in these properties produce inconsistent outcomes and limit reproducibility.^127^ Moreover, hybrid heating is complex due to simultaneous conduction and direct microwave heating of plastics.^127^

A major limitation is the lack of reliable dielectric characterization data for susceptors. Key parameters—dielectric constant, loss factor, loss tangent, and penetration depth—are rarely reported, especially at elevated temperatures, impeding experimental design and material selection. Consequently, a standard susceptor for microwave-assisted plastic pyrolysis has not yet been established, as its selection remains process-specific.

Temperature measurement also remains problematic. Standing wave patterns within microwave fields create strong nonuniformities,^220^ with uniform heating achievable only in sample smaller than the microwave wavelength.^221,222^ Common techniques such as thermocouples, infrared cameras, and optical pyrometers, are prone to inaccuracies,^223,224^ causing discrepancies in reported temperatures,^225^ and hindering scale-up beyond laboratory conditions.^109^

Additionally, interactions between microwaves and plastic/susceptor composites remain poorly understood. Hotspots are the only confirmed microwave-specific effect, while reported anomalous results lack consistent mechanistic explanations. For instance, Cui et al.^149^ found that MAP of PP produced oils rich in cycloalkanes, olefins, and alkanes via random chain scission and β-breakage, whereas Undri et al.^132^ reported mainly methyl-branched hydrocarbons from PP backbone cleavage. The absence of a unified mechanistic framework continues to limit progress and industrial adoption of microwave plastic pyrolysis.

## 9 Summary of the Current State of Research and Existing Gaps in MAPP

This review has demonstrated the effectiveness of MAPP for converting plastic waste into valuable products. The following key insights summarize the current research landscape.

i. MAPP has been applied to both single and mixed plastics for producing oil and gas fractions, with detailed characterization of composition and potential applications. However, most studies have focused on single polymers, with limited work on mixed plastic systems.
ii. The influence of key microwave pyrolysis parameters such as temperature, plastic/susceptor ratios, catalyst type, plastic/catalyst ratios, reaction time, and heating rates on product yields and composition has been widely reported for both single and mixed plastics.
iii. Microwave copyrolysis of plastics with biomass (e.g., cellulose, lignin, rice, wheat straw, bamboo, sugar cane) has been widely explored to enhance oil quality and recovery of valuable chemicals. Studies have investigated how ratios of susceptor/plastic/biomass, temperature, and catalyst selection affect yield and product characteristics.
iv. Hydrogen and CNT recovery from single, mixed, and plastic/biomass systems using various catalysts under microwave-assisted processes has also been widely studied.

Despite these advances, several research gaps remain:

i. Limited comparative studies exist on product distribution and composition between microwave and conventional pyrolysis of single and mixed plastics.
ii. Further work is needed on the effect of catalysts with diverse physicochemical properties—such as variations in metal composition (e.g., Fe, Ni, Co), support material (e.g., Al_2_O_3_, SiO_2_, ZSM-5, biochar), surface area, acidity/basicity, and particle morphology—on MAPP product yield and composition, using standardized parameters (temperature, power, reaction time) and comparing different reactor configurations (e.g., premixed vs nonpremixed systems).
iii. Further knowledge on the real interactions between MW and materials and the changes that occur during heating beyond the reported MW-specific effects—such as non-thermal effects—is essential and worth exploring to unlock the full potential of this emerging technology.
iv. Research on developing carbon-based adsorbents from MAPP char for pollutant removal remains scarce. Applications could target dyes, trace metals, pharmaceuticals, PAHs, antibiotics, and gases (e.g., CO_2_, CO, VOCs), though a few recent studies address microwave activation of conventional char for CO_2_ capture.^71,73^

v. Further studies are required on synthesizing advanced carbon adsorbents via microwave copyrolysis of plastics with nitrogen-rich materials (e.g., melamine, urea, peptone, uric acid) for environmental and energy applications.
vi. Modeling approaches, including machine learning, should be employed to predict pollutant adsorption on MAPP-derived adsorbents.
vii. Comparative energy-efficiency assessments of microwave and conventional pyrolysis under varied conditions are needed.
viii. LCA and techno-economic analyses (TEA) studies are limited. While some studies have examined microwave copyrolysis of plastics with biomass for fuel oils,^226,197^ further work is required to validate environmental and economic feasibility at laboratory and larger scales.

## 10 Conclusion

The conversion of plastic waste into valuable products via pyrolysis is a promising solution to global plastic pollution. Among available methods, MAPP offers distinct advantages, including rapid, selective, and volumetric heating, lower operating temperatures, shorter processing times, and enhanced catalytic efficiency. These features enable the recovery of high-quality fuel-like oils and carbon-rich char with properties comparable to fossil-derived products. Product yield and composition depend on process parameters and plastic type. Owing to these benefits, MAPP is considered a superior alternative to conventional pyrolysis. However, as an emerging technology, it still faces technical and scalability challenges that must be addressed to fully realize its industrial potential, as discussed throughout this review.

## Supplementary Material



## Notes

Summary of conventional plastic recycling methods like mechanical, chemical, thermal, and thermochemical, and their key advantages and limitations; CO_2_ adsorption capacities of plastic-derived activated carbons, emphasizing effects of feedstock, activation, and testing conditions; catalytic pyrolysis studies, detailing operating parameters, catalyst systems, and product yields; MAP studies for single, mixed, and copyrolysis systems, summarizing product yields and oil compositions; catalyst effects on MAP of single plastics; composition and properties of MAP-derived pyro-oils, including heating value, density, viscosity, and key compounds such as aromatics, olefins, and phenolics (PDF)
