# Supplementary material for: Plastic Valorization into Added-Value Products via Microwave and Conventional Pyrolysis: A Review
Source: ACS Environ Au. 2026 Jan 6;6(2):174–95. doi: 10.1021/acsenvironau.5c00077 (PMC13003366; doi:10.1021/acsenvironau.5c00077)
Supplement: Supplementary file 1 [file vg5c00077_si_001.pdf]

Supplementary information

Plastic valorisation into added-value products via microwave and conventional pyrolysis: a review

Emmanuel Dan[†], Alan J McCue[‡], Davide Dionisi[†], Claudia Fernández Martín[*, †]

[†] School of Engineering, Chemical Processes and Materials Engineering Group, University of Aberdeen, Aberdeen AB24 3UE, United Kingdom

[‡] Advanced Centre for Energy and Sustainability (ACES), Department of Chemistry, University of Aberdeen, AB24 3UE, United Kingdom

*Corresponding author: cfmartin@abdn.ac.uk

Table S1: Conventional plastic recycling methods

Recycling method	Examples	Advantages	Challenges
Mechanical	Sorting, shredding, remelting	Reduces plastic landfill waste	Contamination limits efficiency, and cost-ineffective *[1]*, Mechanical performance of recycled plastics declines with multiple recycling cycles *[2]*. Recycled plastics possess reduced tensile strength, elasticity, and durability due to degradation of the polymer chain*[3]*, hence are known to be of low quality.
Chemical	Depolymerisation, solvolysis	High purity recovered materials	Expensive and complex in operation *[4]*, Require purification of final product *[3]*, sensitivity to reaction conditions like temperature and catalyst *[5]*, catalyst efficiency and selectivity issues *[6]*, inherent energy and equilibrium constraints that affect the efficiency and feasibility of breaking down polymers, implying high temperatures or the need of a catalyst *[6]*.

Thermal	Incineration	Energy recovery	Air pollution resulting in human risk *[7]*, Emission of harmful gases (e.g. dioxins, furans) *[8]*, plastic only changes form (plastics are only burned and doesn't disappear or vanish) *[9]*, only beneficial under very efficient energy recovery systems *[10]*.
Thermochemical	Pyrolysis,<br><br>gasification, hydrothermal treatment	High-value product output	Energy-intensive, high costs *[4]*, Pyrolysis requires high temperatures (300–900 °C), especially for gas production, increasing energy demand and costs *[11]*. Optimal heat and mass transfer requires complex reactor configurations (e.g., fluidized beds, semi-batch systems) *[12]* . Material deposition (e.g., coke or residue) on catalyst surface during pyrolysis reduces activity, requiring frequent regeneration or replacement *[13]*. Some effective catalysts (e.g., Pt, Pd, Ru-based) are expensive and may not be practical for large-scale use without cost-efficient regeneration methods *[14]*.<br>Gasification sometimes results in tar formation which causes operational difficulties such as clogging, fouling of equipment, and reduced efficiency of gas cleaning systems. High tar content limits the usability of the produced syngas in downstream applications *[15]*

			High gas flow rates during gasification can dilute the syngas, leading to lower calorific value and making separation of useful gases more difficult *[15]*.<br><br>Hydrothermal treatment (HT) produces liquid effluents containing organics, acids, and halides, necessitating further treatment *[16]* since the plastics undergo thermal and hydrolytic cleavage. This releases oxygenated organics such as carboxylic acids, alcohols, and ketones into the aqueous phase *[17]*. Effective HT often requires supercritical conditions (e.g., >374 °C, >22 MPa), making reactor design and operation complex and energy-intensive *[18]*. Catalysts or pre-treatment are required for HT of some plastics like PE and PP, to improve conversion *[17]*.

Table S2: CO_2 adsorption capacity of reported plastic-based ACs produced from conventional pyrolysis

Plastic type	Modi-fication	Adsorbent type	Activation agent/temp (°C)	Adsorption temperature (°C)/pressure (bar)	CO_2 adsorption (mmol/g)	Ref
PET	-	O-enriched carbon	KOH/ 700	30/1	1.31	[19]
PET	-	O-enriched carbon	KOH/ 800	30/1	1.50	[19]
PET	-	AC	CO_2/925	25/4	6.59	[20]
PET	-	AC	KOH/700	25/1	3.87	[21]
PET	Urea	NPC	KOH/700	25/1	4.58	[21]
PET	-	AC	KOH/700	25/1	0.004	[21]
PET	-	AC	KOH/1000	25/1	0.003	[21]
PET	-	AC	NaOH/700	25/1	0.004	[21]
PET	-	AC	NaOH/1000	25/1	0.024	[21]

PET	-	AC	KOH/525	25/1	0.05	[22]
PET	Acridine	NPC	KOH/525	25/1	0.07	[22]
PET	Carbazole	NPC	KOH/525	25/1	0.11	[22]
PET	Urea	NPC	KOH/525	25/1	0.06	[22]
PET	Melamine/ eutectic salt	NPC	-/550	25/1	6.47	[23]
PET	-	AC	CO_2/925	25/1	2.30	[24]
PET	-	AC	KOH/800	25/1	3.50	[25]
PET	-	AC	KOH/975	25/1	1.42	[26]
PET	Dolomite/ MgO	AC	-/700	20/1	0.22	[27]
PVC	TBAB	AC	-/-	25/1	5.47	[28]
PS	CCl4	AC	-/-	25/1	1.47	[28]
PS	BCMB	AC	-/-	25/1	1.36	[28]
PS	DPX	AC	-/-	25/1	1.28	[28]
PS	FDA	AC	-/-	25/1	1.21	[28]
Mixed plastic	OMMT	AC	KOH/850	25/4	6.75	[29]
Mixed plastic	-	AC	KOH/800	25/1	1.41	[30]
Mixed plastic	-	AC	KOH/700	25/1	1.97	[31]

OMMT – organically modified montmorillonite, TBAB - tetrabutylammonium bromide, BCMB – 4,4'-bis (chloro methyl)-1,1'-biphenyl, DPX – α, α'-dibchloro-p-xylene, FDA – formaldehyde dimethyl acetal, NPC – nitrogen porous carbon, PCNS – porous carbon nanosheet, AC – activated carbon, (-) means nil

Table S3: Catalytic pyrolysis of plastics using conventional reactors

Process information	Product yield						
Plastic	Reactor	Catalyst/ support	Pyrol/ Cat. Temp (°C)	H_2 (mmol/g)	solid (wt.%)	Liquid (wt.%)	Ref
HDPE	Multi-core	Ni-Mo/MgO	600/800	-	16.30	46.21	[32]
HDPE	Muffle furnace	Ni/-	600/800	2.10	24.50	10.20	[33]
PP	FBR	Fe/Al_2O_3	500/800	24.18	30.20	18.11	[34]
HDPE	FBR	Fe/Al_2O_3	500/800	24.30	36.91	20.00	[34]
LDPE	FBR	Fe/Al_2O_3	500/800	20.75	35.90	20.70	[34]
HIPS	FBR	Fe/Al_2O_3	500/800	13.14	49.40	20.80	[34]
GPPS	FBR	Fe/Al_2O_3	500/800	12.21	48.71	26.12	[34]
PS	Muffle furnace	Natural zeolite	450/500	-	60.00	24.61	[35]
PP	Muffle furnace	Natural zeolite	450/500	-	54.10	19.90	[35]
PE	Muffle furnace	Natural zeolite	450/500	-	42.12	7.21	[35]

PP	FBR	Ni-Mn/Al_2O_3	500/800	56.61	-	46.60	[36]
HDPE	Fluidized bed/CSBR	Ni/Al_2O_3 in Ca	500/900	183.65	-	-	[37]
LDPE	Semi-batch	Ni/Al_2O_3 in Ca	500/800	190.50	-	-	[38]
PP	Semi-batch	Fe/Al_2O_3	500/800	31.07	5.70	63.23	[38]
LDPE	Semi-batch	-/-	500/800	18.20	-	-	[38]
PP	FBR	Fe-small/-	600/800	15.40	26.01	-	[39]
PP	FBR	Fe-large/-	600/800	25.60	29.10	-	[39]
PP	FBR	Ni-small/-	600/800	18.20	16.00	-	[39]
PP	FBR	Ni-large/-	600/800	22.60	16.00	-	[39]
HDPE	CSBR/Fluidized bed	Ni-based/Al_2O_3	500/700	186.50	-	-	[40]
PP	FBR	-	-	174.00	-	-	[41]
HDPE	FBR	Ni-Mg-Al	500/800	20.10	20.00	-	[41]
PP	FBR	Ni-Zn-Al/-	500/800	45.90	10.00	-	[42]
PP	FBR	Ni-Mg-Al/-	500/800	75.40	3.50	-	[42]
PP	FBR	Ni-Ca-Al/-	500/800	68.50	9.20	-	[42]
PP	FBR	Ni-Ce-Al/-	500/800	63.10	8.00	-	[42]
PP	FBR	Ni-Mn-Al/-	500/800	71.40	23.00	-	[42]
PP/PE	Fluidized bed/FBR	H-Ni/-	500/750	28.61	30.50	-	[43]
PP/PE	Fluidized bed/FBR	H-Ni/-	500/600	25.50	20.00	-	[43]
PP	FBR	Ni_2O_3/-	500/830	-	7.80	-	[44]
PP	FBR	Ni $(OH)_2$/-	500/830	-	55.60	-	[44]
PP	FBR	NiO/-	500/830	-	13.60	-	[44]
PP	FBR	$NiCO_3$.2Ni $(OH)_2$/-	500/830	-	49.50	-	[44]
LDPE	FBR	Ni/Al_2O_3	600/800	36.60	42.10	-	[45]
LDPE	FBR	Fe/Al_2O_3	600/800	25.20	36.20	-	[45]
LDPE	FBR	Co/Al_2O_3	600/800	31.10	28.10	-	[45]
LDPE	FBR	Cu/Al_2O_3	600/800	26.00	24.00	-	[45]
HDPE	FBR	ZSM5-30	500/800	57.40	-	-	[46]
HDPE	FBR	β-zeolite-25/-	500/800	52.87	-	-	[46]
HDPE	FBR	ɣ-zeolite-30/-	500/800	55.42	-	-	[46]
HDPE	FBR	Ni/ZSM5-30	500/800	66.09	-	-	[46]
HDPE	FBR	Ni/β-zeolite-25	500/800	61.38	-	-	[46]
HDPE	FBR	Ni/ɣ-zeolite-30	500/800	58.06	-	-	[46]
HDPE	CSBR/FBR	Ni/Al_2O_3	500/700	34.50	4.45	-	[47]
LDPE	FBR	Co/MgO	500/500	-	29.40	-	[48]

LDPE	FBR	Co-Mo/MgO	500/500	-	10.50	-	[48]
PP	Autoclave	Ferrocene-sulphur	700/800	-	62.80	-	[49]
PP	Tubular	Nickel Foam/-	500/800	-	43.12	-	[50]
PS	Tubular	Ni-Fe/Al_2O_3	600/800	-	29.5	-	[51]
HDPE	Batch	HZSM-5	-	-	-	45.1	[52]
Mixed plastic	FBR	Ni-Fe_{13}/Al_2O_3	500/800	42.00	50.09	8.80	[43]
Mixed plastic	FBR	Ni-Fe_{12}/Al_2O_3	500/800	38.80	49.90	13.50	[43]
Mixed plastic	FBR	Ni-Fe_{11}/Al_2O_3	500/800	34.52	45.80	7.11	[43]
Mixed plastic	FBR	Ni-Fe_{21}/Al_2O_3	500/800	33.87	45.10	9.90	[43]
Mixed plastic	FBR	Ni-Fe_{31}/Al_2O_3	500/800	36.20	45.80	10.30	[43]
Mixed plastic	FBR	Ni/ɣ-Al_2O_3	500/800	18.00	26.10	-	[43]
Mixed plastic	FBR	Fe/ɣ-Al_2O_3	500/800	22.90	32.60	-	[43]
Mixed plastic	FBR	Fe/α-Al_2O_3	500/800	20.70	35.20	-	[43]
Mixed plastic	FBR	Ni/α-Al_2O_3	500/800	22.50	21.10	-	[43]
Mixed plastic	FBR	Ni-Fe/ɣ-Al_2O_3	500/800	31.80	40.70	-	[43]

*FBR – fixed bed reactor; CSBR - conical spouted bed reactor, dash (-) means not found

Table S4: Microwave-assisted pyrolysis of single plastics

			Product yield (wt.%)				
Plastic type	Susceptor/ catalyst	Power (kW)	Solid	Liquid	Gas	Oil composition /Remark	Ref
PS	carbon	3	1	92.3	6.7	Oil contained styrene and aromatics	*[53]*
PVC	AC	-	8.1	70	22	High quality oil	*[54]*
PP	Spherical AC	0.9	3	72	25	Light oil	*[55]*
PS	AC	0.45	10	77	12	Oil contains C_{13} and aromatics	*[56]*
PP	-	0.75	5.4	1.1	93.5	-	*[15]*
PP	Graphite	0.45	1.18	48.16	50.66	Oil of high heating value	*[57]*
PP	AC	0.45	0.4	29.8	69.8	Oil of high heating value	*[57]*

PP	SiC	0.45	1.59	31.89	66.52	Oil of high heating value	*[57]*
PP	Al	0.45	1.18	45.4	53.42	Oil of high heating value	*[57]*
PP	Fly-ash	0.45	0.7	21.07	78.23	Oil of high heating value	*[57]*
LDPE	Fe powder	0.70	0.20	39.98	44.50	1-Decene, Cyclohexane,1,2,3,5-tetraisopropyl, 1-Undecene	*[58]*
PS	Fe powder	0.70	0.10	76.79	21.31	Styrene, Benzene, 3-butynyl	*[58]*
LDPE	Graphite	0.45	0.8	46.50	52.7	Oil of high heating value	*[57]*
PP	Graphite	0.6	1.5	46.3	52	High quality oil composed of aromatics, HCs, oxygenates.	*[59]*
PS	Graphite	0.6	1	78.2	20.8	High quality oil composed of aromatics, HCs, oxygenates.	*[59]*
PS	Carbon	3	0.9	96.1	3	Low viscous oil.	*[60]*
PS	Tire	3	31.8	57.4	10.8	Low viscous oil.	*[60]*

Table S5: Microwave-assisted pyrolysis of mixed plastics

			Product yield (wt.%)				
Plastic type	Susceptor/ catalyst	Power (kW)	Solid	Liquid	Gas	Oil composition /Remark	Ref
PP/PS/LDPE	Fe-powder/Fe-coil	0.70	-	69.24	-	Oil contained aliphatic and aromatics	*[58]*
PS/PP	Fe coil	0.5-2.5	-	65.67	-	Styrene, benzene, 3-nitropropyl, 2,4 dimethyle-1-heptene	*[61]*
PS/LDPE	Fe-coil	0.5-2.5	-	46.52	-	Toluene, 1,3,5,7-Cyclooctatetraene, alpha-Methyl styrene, Benzenebutanenitrile	*[61]*
PS/PP/LDPE	Fe-coil	0.5-2.5	-	57.23	-	2,4-Dimethyl-1-heptene, styrene	*[61]*

PP/PS	Rice husk-AC	0.95	1.22	69.55	21.23	High quality oil	*[62]*
PP/PS	Coconut sheet-AC	0.95	-	77.4	22.6	High quality oil	*[62]*
PP/PS	Corn husk-AC	0.95	-	84.30	15.7	High quality oil	*[62]*
PP/PS	Rice husk-carbon	0.95	1.4	50.65	49.75	High quality oil	*[62]*
PP/PS	Coconut sheet-carbon	0.95	0.2	58.05	41.75	High quality oil	*[62]*
PP/PS	Corn husk-carbon	0.95	-	81.5	18.5	High quality oil	*[62]*
PET/LDPE	Coconut husk AC	-	-	51.67	33.33	Oil composed mainly monocyclic aromatics	*[63]*

Table S6: Microwave-assisted co-pyrolysis

			Product yield (wt.%)				
Plastic/ Biomass	Susceptor/ catalyst	Power (kW)	Solid	Liquid	Gas	Oil composition /Remark	Ref
PVC/micro-algae	AC	-	17	55	27	Improve oil quality than without microalgae	*[54]*
PP/lignin	SiC/ HZSM-5	1	15	56	25	High quality bio-oil	*[64]*
LDPE/Lignin	SiC/ HZSM-5	0.75	15	41.2	35.9	Oil contains phenol, aromatics and HCs	*[65]*
LDPE/sugar-cane bagasse	HZSM-5	0.5	17.65	43.48	38.21	High quality bio-oil containing alkene, oxygenates and naphthalene	*[66]*
LDPE/Rice straw	HZSM-5	0.75	16.55	39.18	44.27	Bio-oil contain HCs, ketone, ester, and phenols	*[67]*

LDPE/lignin	AC	0.75	10	36	54	Oil contains aldehydes, ketones, furans	[68]
HDPE/used cooking oil	AC	0.8	1-6	61-84	15-41	Oil composed oxygenates, benzene, and HCs	[69]
HDPE/used frying oil	-	0.75	1	81	18	Oil with commercial diesel properties	[70]
HDPE/bamboo	AC/Ni	0.3	8.6	10.3	81.1	-	[71]
PP/chili straw	HZSM-5	0.57	20	58	22	Oil rich in HCs	[72]
PP/bam-boo	HZSM-5	1	2	64	28	Oil with jet fuel properties	[73]
PP/wheat straw	Graphite	0.6	7.8	55.6	36.6	High quality oil composed of aromatics, HCs, oxygenates.	[59]
PP/rice husk	Graphite	0.6	10.1	51.5	38.4	High quality oil composed of aromatics, HCs, oxygenates.	[59]
PS/wheat straw	Graphite	0.6	8.4	64.9	26.7	High quality oil composed of aromatics, HCs, oxygenates.	[59]
PS/rice husk	Graphite	0.6	10.9	61.7	-	High quality oil composed of aromatics, HCs, oxygenates.	[59]

Table S7: Effect of catalyst on microwave pyrolysis of LDPE

Catalyst	Pyrol. /Cat. temp (°C)	Yield (wt.%) Oil/gas/solid	Remark	Role of catalyst	Ref

NiO, HY-zeolite	500/450	56.53/-/-	Aromatic and aliphatic compounds in the oil increases from 23.5 – 80.40% in the presence of HY.	HY converts light gases into larger molecules. NiO promotes the breakdown of large molecules into smaller radicals	*[74]*
HZSM-5	700/550	42/46/-	Oil composed of paraffin and PAHs (90% of aromatics)	HZSM-5 aid the cracking of alkane to aliphatic hydrocarbon through oligomerization	*[75]*
Bentonite	700/500	87.6/-/-	Oil fraction composed of 3.59% (C_5-C_9) aromatic, 22.28% (C_{10}-C_{13}) non-aromatic, and 62.08% > C_{13}	Bentonite eliminated pressure drop and reduce the reaction time	*[76]*
ZSM-5	480/450	32.58/-/-	Oil contained mono-ring aromatic hydrocarbon (74.73-88.49%) in the oil. Syngas composed of light olefins (C_2), paraffin, and H_2	ZSM-5 favour H-removal from the gas fraction resulting in increased H_2 production	*[77]*
Natural zeolite	550/550	-/28.12/ 2.88	The liquid composed 19.02 % of hydrocarbons including aromatics that were absence in the uncatalyzed liquid fraction	Zeolite promoted LDPE degradation and increase the liquid yield	*[78]*
MgO	> 500/450	31.35/56.5/ <7.1	0.14-1.9 wt. % coke. Monoaromatics and C_5-C_{12} aliphatic in the oil increase from 10–30% with catalysis, while the gas contains H_2, C_1-C_3 olefins, and paraffins	MgO aided the breaking down of long chain hydrocarbons to shorter ones.	*[79]*

Ru/ ZSM-5	300/300	2/88/-	Gas yield reaches 90 wt.%, with 23 wt.% aromatics selectivity and 30 wt.% light olefins selectivity.	Ru/ ZSM-5 provides active sites for catalytic reaction	*[80]*

Table S8: Effect of catalyst on microwave pyrolysis of HDPE

Catalyst	Pyrol. /Cat. temp (°C)	Yield (wt.%) Oil/gas/solid	Remark	Role of catalyst	Ref
Bentonite	700/500	88.7/-/-	Oil fraction composed of 3.12% (C_5-C_9) aromatic, 21.43% (C_{10}-C_{13}) non-aromatic, and 62.73% > C_{13}	Bentonite eliminated pressure drop and reduce the reaction time	*[76]*
Activated carbon and molecular sieve (13X & MS4A)	-/-	82.35/-/-	Oil contains hydrocarbons with carbon number mainly between C_7 and C_{20}	AC promoted wax formation. Catalyst also enhances the breaking of long hydrocarbons and shorten the whole reaction time.	*[81]*
ZSM-5	620/400	48.9/-/-	73.5% gasoline-range hydrocarbons rich in aromatic (45.0%) and isomerized aliphatic (24.6%) contents	ZSM-5 enabled the elimination of wax product and increases the liquid yield	*[82]*
Al_2O_3-$ZnFe_2O_4$	450/450	7.5/19.5/ 71.5	Al_2O_3-$ZnFe_2O_4$ resulted in the least carbon deposition, showing high H_2 yield and low oil formation	Al_2O_3-$ZnFe_2O_4$ increased solid and gas yields, reduced energy of reaction.	*[83]*
$NiFe_2O_4$	400/400	-/-/73	93 wt.% (57 mmol/g) of H_2 recovered from the gas	$NiFe_2O_4$ suppressed the formation of tar and char. Also,	*[84]*

			fraction. Gas contained CH_4, C_2H_4, C_2-C_5, CO and CO_2.	promotes the formation of light gasses	
Fe/Ni-CeO_2@CNTs	-/800	-/-/-	Gas fraction yielded 50.2 mmol/g plastic of H_2.	Fe/Ni-CeO_2@CNTs promoted H2 yield and increase selectivity	*[85]*
SiC	460/-	71/29/0	Oil composed of C_8-C_{16} hydrocarbons	-	*[86]*

Table S9: Chemical and physical properties of pyro-oil from MAPPs

Plastic type/ modification	Pyrolysis conditions	Some Specific Compounds in oil fraction	Physical properties of bio-oil				
			HHV (MJ/kg)	Den-sity (g/cm^3)	Visco-sity (cSt)	API	Refs
PS/-	Carbon, 3.00 kW, 2.45 GHz	2-butene, 1,3-pentadiene, benzene, toluene, 3-methyl -1-hexene, ethyl benzene, styrene, 1,4-dimethyl benzene, cumene, limonene, l,3-diphenyl propane, 2-phenyl naphthene	40	0.92	0.91	25	[60]
PS/-	Tire, 3.00kW, 2.45 GHz	Similar oil composition obtained with carbon absorber but with varied amount.	41	0.89	0.79	22	[60]
PP/Lignin	SiC/HZSM-5, 0.75kW, 2.45GHz	Methyl phenol, methanol, methoxy phenol, guaiacols, syringols, napthalenes	-	-	-	-	[65]
LDPE/-	ZSM-5, 1kW, 2.45GHz	Xylenes, ethyltoluene, trimethylbenzene, indane, diethylbenzene, naphthalene, methylnaphthalen, dimethylnaphthalene, vinylnaphthalene	45	-	-	-	[87]

PS/-	AC, 0.45kW, 2.45GHz	Propane, 2-methyl butane, cyclohexane, hexane, cyclobutene, benzene, butene, methyl benzene	45	0.90	1.67	28	[56]
PS/-	Iron mesh, 0.75kW, 2.45GHz	4-Chloro-1,2,3,4-tetrahydro-4-methylnaphthalen-1-ol, 1-Hydroxy-4-chloronapthalene, ethylbenzene, anthrol, 2,5-Dimethyl-1,3-bis(2-phenyl(ethyl)benzene, vinyl-anthracene	41	-	0.80	26	[88]
PS/-	Carbon, 3kW, 2.45GHz	Benzene, toluene, phenylethane, styrene, cumene, mesitylene, allylbenzene, methylstyrene, 1-Methyl-2-vinylbenzene, (E)-Prop-1-enylbenzene, naphthalene, indene, 2,3-Diphenylbutane	-	0.80 – 0.92	0.64 – 1.04	-	[53]
PS/-	SiC, 3kW, 2.45GHz	Benzene, toluene, phenylethane, styrene, cumene, mesitylene, allylbenzene, methylstyrene, 1-Methyl-2-vinylbenzene, (E)-Prop-1-enylbenzene, naphthalene, indene, 2,3-Diphenylbutane	-	0.92	1.13	-	[53]
PS, PP, PE/-	Fe metal powder and Fe-coil	Styrene, 2,4-Dimethyl-1-heptene, 2,4-Dimethyl-1-decene, 1-Decene, 1-Undecene, 2,3,7-Trimethyl-2-octene	45.40	0.83	-	-	[58]

PP/-	Fe metal powder and Fe-coil	Styrene, 1-Octene, Ethylbenzene, 2,4-Dimethyl-1-heptene, 2,4-Dimethyl-1-decene, 1-Decene, 1-Undecene, 2,3,7-Trimethyl-2-octene,	46.52	0.88	-	-	[58]

*API – American Petroleum Institute, gravity

References

[1] Serranti S, Bonifazi G. Techniques for separation of plastic wastes. Use of Recycled Plastics in Eco-efficient Concrete, Elsevier; 2018, p. 9–37. https://doi.org/10.1016/B978-0-08-102676-2.00002-5.

[2] Parameswaranpillai J, Mavinkere S, Arpitha R, Rajkumar G, Siengchin S. Composites Science and Technology Recent Developments in Plastic Recycling. Singapore: 2021. https://doi.org/https://doi.org/10.1007/978-981-16-3627-1.

[3] Sambyal P, Najmi P, Sharma D, Khoshbakhti E, Hosseini H, Milani AS, et al. Plastic recycling: Challenges and opportunities. Canadian Journal of Chemical Engineering 2024. https://doi.org/10.1002/cjce.25531.

[4] Huang J, Veksha A, Chan WP, Giannis A, Lisak G. Chemical recycling of plastic waste for sustainable material management: A prospective review on catalysts and processes. Renewable and Sustainable Energy Reviews 2022;154. https://doi.org/10.1016/j.rser.2021.111866.

[5] Jiang Z, Liang Y, Guo F, Wang Y, Li R, Tang A, et al. Microwave-Assisted Pyrolysis-A New Way for the Sustainable Recycling and Upgrading of Plastic and Biomass: A Review. ChemSusChem 2024;17. https://doi.org/10.1002/cssc.202400129.

[6] Payne J, Jones MD. The Chemical Recycling of Polyesters for a Circular Plastics Economy: Challenges and Emerging Opportunities. ChemSusChem 2021; 14:4041–70. https://doi.org/10.1002/cssc.202100400.

[7] Okunola A A, Kehinde I O, Oluwaseun A, Olufiropo E A. Public and Environmental Health Effects of Plastic Wastes Disposal: A Review. Journal of Toxicology and Risk Assessment 2019;5. https://doi.org/10.23937/2572-4061.1510021.

[8] Nagy Á, Kuti R. The Environmental Impact of Plastic Waste Incineration. Academic and Applied Research in Military and Public Management Science 2016; 15:231–7. https://doi.org/10.32565/aarms.2016.3.3.

[9] ManishaaKSangalee M. Bioremediation Technology for Plastic Waste. Singapore: 2019. https://doi.org/https://doi.org/10.1007/978-981-13-7492-0.

[10] Eriksson O, Finnveden G. Plastic waste as a fuel - CO_2-neutral or not? Energy Environ Sci 2009; 2:907–14. https://doi.org/10.1039/b908135f.

[11] Al-Salem SM. Thermal pyrolysis of high-density polyethylene (HDPE) in a novel fixed bed reactor system for the production of high value gasoline range hydrocarbons (HC). Process Safety and Environmental Protection 2019; 127:171–9. https://doi.org/10.1016/j.psep.2019.05.008.

[12] Ko S, Kwon YJ, Lee JU, Jeon YP. Preparation of synthetic graphite from waste PET plastic. Journal of Industrial and Engineering Chemistry 2020; 83:449–58. https://doi.org/10.1016/j.jiec.2019.12.018.

[13] Li Q, Faramarzi A, Zhang S, Wang Y, Hu X, Gholizadeh M. Progress in catalytic pyrolysis of municipal solid waste. Energy Convers Manag 2020;226. https://doi.org/10.1016/j.enconman.2020.113525.

[14] Singh RK, Ruj B, Sadhukhan AK, Gupta P. Impact of fast and slow pyrolysis on the degradation of mixed plastic waste: Product yield analysis and their characterization. Journal of the Energy Institute 2019; 92:1647–57. https://doi.org/10.1016/j.joei.2019.01.009.

[15] Jiang L, Zhou Z, Xiang H, Yang Y, Tian H, Wang J. Characteristics and synergistic effects of co-pyrolysis of microalgae with polypropylene. Fuel 2022;314. https://doi.org/10.1016/j.fuel.2021.122765.

[16] Ling M, Ma D, Hu X, Liu Z, Wang D, Feng Q. Hydrothermal treatment of polyvinyl chloride: Reactors, dechlorination chemistry, application, and challenges. Chemosphere 2023;316. https://doi.org/10.1016/j.chemosphere.2022.137718.

[17] Xu Q, Wang Q, Yang J, Liu W, Wang A. Recovering Valuable Chemicals from Polypropylene Waste via a Mild Catalyst-Free Hydrothermal Process. Environ Sci Technol 2024; 58:16611–20. https://doi.org/10.1021/acs.est.4c04449.

[18] Ozoemena MC, Coles SR. Hydrothermal Treatment of Waste Plastics: An Environmental Impact Study. J Polym Environ 2023; 31:3120–30. doi.org/10.1007/s10924-023-02792-3.

[19] Kaur B, Gupta RK, Bhunia H. Chemically activated nanoporous carbon adsorbents from waste plastic for CO_2 capture: Breakthrough adsorption study. Microporous and Mesoporous Materials 2019; 282:146–58. https://doi.org/10.1016/j.micromeso.2019.03.025.

[20] Moura PAS, Vilarrasa-Garcia E, Maia DAS, Bastos-Neto M, Ania CO, Parra JB, et al. Assessing the potential of nanoporous carbon adsorbents from polyethylene terephthalate (PET) to separate CO_2 from flue gas. Adsorption 2018; 24:279–91. https://doi.org/10.1007/s10450-018-9943-4.

[21] Yuan X, Gyu J, Yun H, Deng S, Jin Y, Eun J, et al. Solving two environmental issues simultaneously: Waste polyethylene terephthalate plastic bottle-derived microporous carbons for capturing CO_2. Chemical Engineering Journal 2020; 397:125350. https://doi.org/10.1016/j.cej.2020.125350.

[22] Arenillas A, Rubiera F, Parra JB, Ania CO, Pis JJ. Surface modification of low-cost carbons for their application in the environmental protection 2005; 252:619–24. https://doi.org/10.1016/j.apsusc.2005.02.076.

[23] Song C, Zhang B, Hao L, Min J, Liu N, Niu R, et al. Converting poly (ethylene terephthalate) waste into N-doped porous carbon as CO_2 adsorbent and solar steam generator. Green Energy and Environment 2022; 7:411–22. https://doi.org/10.1016/j.gee.2020.10.002.

[24] Parra JB, Ania CO, Arenillas A, Rubiera F, Palacios JM, Pis JJ. Textural development and hydrogen adsorption of carbon materials from PET waste. J Alloys Compd 2004; 379:280–9. https://doi.org/10.1016/j.jallcom.2004.02.044.

[25] Adibfar M, Kaghazchi T, Asasian N, Soleimani M. Conversion of Poly (Ethylene Terephthalate) Waste into Activated Carbon: Chemical Activation and Characterization. Chem Eng Technol 2014; 37:979–86. https://doi.org/10.1002/ceat.201200719.

[26] Esfandiari A, Kaghazchi T, Soleimani M. Preparation and evaluation of activated carbons obtained by physical activation of polyethylene terephthalate (PET) wastes. J Taiwan Inst Chem Eng 2012; 43:631–7. https://doi.org/10.1016/j.jtice.2012.02.002.

[27] Przepiórski J, Czyzewski A, Pietrzak R, Morawski AW. MgO/CaO-loaded activated carbon for carbon dioxide capture: Practical aspects of use. Ind Eng Chem Res 2013; 52:6669–77. https://doi.org/10.1021/ie302848r.

[28] Fu Z, Mohamed IMA, Li J, Liu C. Novel adsorbents derived from recycled waste polystyrene via cross-linking reaction for enhanced adsorption capacity and separation selectivity of CO_2. J Taiwan Inst Chem Eng 2019; 97:381–8. https://doi.org/10.1016/j.jtice.2019.01.014.

[29] Gong J, Michalkiewicz B, Chen X, Mijowska E, Liu J, Jiang Z, et al. Sustainable conversion of mixed plastics into porous carbon nanosheets with high performances in uptake of carbon dioxide and storage of hydrogen. ACS Sustain Chem Eng 2014; 2:2837–44. https://doi.org/10.1021/sc500603h.

[30] Ligero A, Calero M, Pérez A, Solís RR, Muñoz-Batista MJ, Martín-Lara MÁ. Low-cost activated carbon from the pyrolysis of post-consumer plastic waste and the application in CO_2 capture. Process Safety and Environmental Protection 2023; 173:558–66. https://doi.org/10.1016/j.psep.2023.03.041.

[31] Dan E, McCue AJ, Dionisi D, Martín CF. The role of the activation heating source on the carbon capture performance of two new adsorbents produced from household-mixed-plastic waste. Journal of CO2 Utilization 2024;89. https://doi.org/10.1016/j.jcou.2024.102950.

[32] Bajad GS, Vijayakumar RP, Gupta AG, Jagtap V, Singh Y pal. Production of liquid hydrocarbons, carbon nanotubes and hydrogen rich gases from waste plastic in a multi-core reactor. J Anal Appl Pyrolysis 2017; 125:83–90. https://doi.org/10.1016/j.jaap.2017.04.016.

[33] Bazargan A, McKay G. A review - Synthesis of carbon nanotubes from plastic wastes. Chemical Engineering Journal 2012;195–196:377–91. https://doi.org/10.1016/j.cej.2012.03.077.

[34] Cai N, Li X, Xia S, Sun L, Hu J, Bartocci P, et al. Pyrolysis-catalysis of different waste plastics over Fe/Al_2O_3 catalyst: High-value hydrogen, liquid fuels, carbon nanotubes and possible reaction mechanisms. Energy Convers Manag 2021;229. https://doi.org/10.1016/j.enconman.2020.113794.

[35] Miandad R, Rehan M, Barakat MA, Aburiazaiza AS, Khan H, Ismail IMI, et al. Catalytic pyrolysis of plastic waste: Moving toward pyrolysis based biorefineries. Front Energy Res 2019;7. https://doi.org/10.3389/fenrg.2019.00027.

[36] Wu C, Nahil MA, Miskolczi N, Huang J, Williams PT. Production and application of carbon nanotubes, as a co-product of hydrogen from the pyrolysis-catalytic reforming of waste plastic. Process Safety and Environmental Protection 2016; 103:107–14. https://doi.org/10.1016/j.psep.2016.07.001.

[37] Barbarias I, Artetxe M, Lopez G, Arregi A, Bilbao J, Olazar M. Influence of the conditions for reforming HDPE pyrolysis volatiles on the catalyst deactivation by coke. Fuel Processing Technology 2018; 171:100–9. https://doi.org/10.1016/j.fuproc.2017.11.003.

[38] Ahmad I, Khan MI, Khan H, Ishaq M, Tariq R, Ahmad W. Pyrolysis Study of Polypropylene and Polyethylene into Premium Oil Products Pyrolysis Study of Polypropylene and Polyethylene Into Premium Oil Products. Int J Green Energy 2015; 12:663–71. https://doi.org/10.1080/15435075.2014.880146.

[39] Liu X, Zhang Y, Nahil MA, Williams PT, Wu C. Development of Ni- and Fe- based catalysts with different metal particle sizes for the production of carbon nanotubes and hydrogen from thermo-chemical conversion of waste plastics. J Anal Appl Pyrolysis 2017; 125:32–9. https://doi.org/10.1016/j.jaap.2017.05.001.

[40] Sharma A, Kodgire P, Kachhwaha SS. Biodiesel production from waste cotton-seed cooking oil using microwave-assisted transesterification: Optimization and kinetic modelling. Renewable and Sustainable Energy Reviews 2019;116. doi.org/10.1016/j.rser.2019.109394.

[41] Saad JM, Nahil MA, Williams PT. Influence of process conditions on syngas production from the thermal processing of waste high density polyethylene. J Anal Appl Pyrolysis 2015; 113:35–40. https://doi.org/10.1016/j.jaap.2014.09.027.

[42] Nahil MA, Wu C, Williams PT. Influence of metal addition to Ni-based catalysts for the co-production of carbon nanotubes and hydrogen from the thermal processing of waste polypropylene. Fuel Processing Technology 2015; 130:46–53. https://doi.org/10.1016/j.fuproc.2014.09.022.

[43] Yao D, Wu C, Yang H, Zhang Y, Nahil MA, Chen Y, et al. Co-production of hydrogen and carbon nanotubes from catalytic pyrolysis of waste plastics on Ni-Fe bimetallic catalyst. Energy Convers Manag 2017; 148:692–700. https://doi.org/10.1016/j.enconman.2017.06.012.

[44] Jiang Z, Song R, Bi W, Lu J, Tang T. Polypropylene as a carbon source for the synthesis of multi-walled carbon nanotubes via catalytic combustion. Carbon N Y 2007; 45:449–58. https://doi.org/10.1016/j.carbon.2006.08.012.

[45] Acomb JC, Wu C, Williams PT. The use of different metal catalysts for the simultaneous production of carbon nanotubes and hydrogen from pyrolysis of plastic feedstocks. Appl Catal B 2016; 180:497–510. https://doi.org/10.1016/j.apcatb.2015.06.054.

[46] Yao D, Yang H, Chen H, Williams PT. Investigation of nickel-impregnated zeolite catalysts for hydrogen/syngas production from the catalytic reforming of waste polyethylene. Appl Catal B 2018; 227:477–87. https://doi.org/10.1016/j.apcatb.2018.01.050.

[47] Erkiaga A, Lopez G, Barbarias I, Artetxe M, Amutio M, Bilbao J, et al. HDPE pyrolysis-steam reforming in a tandem spouted bed-fixed bed reactor for H_2 production. J Anal Appl Pyrolysis 2015; 116:34–41. https://doi.org/10.1016/j.jaap.2015.10.010.

[48] Aboul-Enein AA, Awadallah AE, Abdel-Rahman AAH, Haggar AM. Synthesis of multi-walled carbon nanotubes via pyrolysis of plastic waste using a two-stage process. Fullerenes Nanotubes and Carbon Nanostructures 2018; 26:443–50. https://doi.org/10.1080/1536383X.2018.1447929.

[49] Li Q, Liu S, Wang L, Chen F, Shao J, Hu X. Efficient nitrogen doped porous carbonaceous CO_2 adsorbents based on lotus leaf. J Environ Sci (China) 2021; 103:268–78. https://doi.org/10.1016/j.jes.2020.11.008.

[50] Eldahshory AI, Emara K, Abd-Elhady MS, Ismail MA. High Quality and Maximizing the Production of CNTs from the Pyrolysis of Waste Polypropylene. Arab J Sci Eng 2023; 48:8655–66. https://doi.org/10.1007/s13369-022-07206-5.

[51] Graves KA, Higgins LJR, Nahil MA, Mishra B, Williams PT. Structural comparison of multi-walled carbon nanotubes produced from polypropylene and polystyrene waste plastics. J Anal Appl Pyrolysis 2022;161. https://doi.org/10.1016/j.jaap.2021.105396.

[52] Usman Azam M, Fernandes A, João Ferreira M, McCue AJ, Graça I, Afzal W. Unlocking the structure-activity relationship of hierarchical MFI zeolites towards the hydrocracking of HDPE. Fuel 2025;379. https://doi.org/10.1016/j.fuel.2024.132990.

[53] Bartoli M, Rosi L, Frediani M, Undri A, Frediani P. Depolymerization of polystyrene at reduced pressure through a microwave assisted pyrolysis. J Anal Appl Pyrolysis 2015; 113:281–7. https://doi.org/10.1016/j.jaap.2015.01.026.

[54] Dai M, Xu H, Yu Z, Fang S, Chen L, Gu W, et al. Microwave-assisted fast co-pyrolysis behaviors and products between microalgae and polyvinyl chloride. Appl Therm Eng 2018; 136:9–15. https://doi.org/10.1016/j.applthermaleng.2018.02.102.

[55] Jing X, Wen H, Gong X, Xu Z. Heating strategies for the system of PP and Spherical Activated Carbon during microwave cracking for obtaining value-added products. Fuel Processing Technology 2020;199. https://doi.org/10.1016/j.fuproc.2019.106265.

[56] Prathiba R, Shruthi M, Miranda LR. Pyrolysis of polystyrene waste in the presence of activated carbon in conventional and microwave heating using modified thermocouple. Waste Management 2018; 76:528–36. https://doi.org/10.1016/j.wasman.2018.03.029.

[57] Suriapparao D V., Vinu R. Resource recovery from synthetic polymers via microwave pyrolysis using different susceptors. J Anal Appl Pyrolysis 2015; 113:701–12. https://doi.org/10.1016/j.jaap.2015.04.021.

[58] Putra PHM, Rozali S, Patah MFA, Ghazali NNN, Ahmad R, Idris A. Effect of metal powder and coil on microwave pyrolysis of mixed plastic. Chemical Engineering Journal 2024;487. https://doi.org/10.1016/j.cej.2024.150530.

[59] Suriapparao D V., Vinu R, Shukla A, Haldar S. Effective deoxygenation for the production of liquid biofuels via microwave assisted co-pyrolysis of agro residues and waste plastics combined with catalytic upgradation. Bioresour Technol 2020;302. https://doi.org/10.1016/j.biortech.2020.122775.

[60] Undri A, Frediani M, Rosi L, Frediani P. Reverse polymerization of waste polystyrene through microwave assisted pyrolysis. J Anal Appl Pyrolysis 2014; 105:35–42. https://doi.org/10.1016/j.jaap.2013.10.001.

[61] Arshad H, Sulaiman SA, Hussain Z, Naz MY, Moni MNZ. Effect of Input Power and Process Time on Conversion of Pure and Mixed Plastics into Fuels Through Microwave-Metal Interaction Pyrolysis. Waste Biomass Valorization 2021; 12:3443–57. https://doi.org/10.1007/s12649-020-01225-9.

[62] Rex P, Masilamani IP, Miranda LR. Microwave pyrolysis of polystyrene and polypropylene mixtures using different activated carbon from biomass. Journal of the Energy Institute 2020; 93:1819–32. https://doi.org/10.1016/j.joei.2020.03.013.

[63] Wang X, Peng Y, Zhou R, Fan L, Zhang Q, Cui X, et al. Production of monocyclic aromatic hydrocarbons from microwave co-pyrolysis of polyethylene terephthalate and low-density polyethylene using coconut husk carbon as microwave absorbent. Chemical Engineering Journal 2024;488. https://doi.org/10.1016/j.cej.2024.150732.

[64] Duan D, Wang Y, Dai L, Ruan R, Zhao Y, Fan L, et al. Ex-situ catalytic co-pyrolysis of lignin and polypropylene to upgrade bio-oil quality by microwave heating. Bioresour Technol 2017; 241:207–13. https://doi.org/10.1016/j.biortech.2017.04.104.

[65] Fan L, Chen P, Zhang Y, Liu S, Liu Y, Wang Y, et al. Fast microwave-assisted catalytic co-pyrolysis of lignin and low-density polyethylene with HZSM-5 and MgO for improved bio-oil yield and quality. Bioresour Technol 2017; 225:199–205. https://doi.org/10.1016/j.biortech.2016.11.072.

[66] Binnal P, Rajashekhara S, Manjunath SP, Ahmed A, Tawaf M, Bhat A. ZSM-5 catalyzed copyrolysis of sugarcane bagasse with LDPE: Influence of microwave-assisted acid pretreatment of sugarcane bagasse on yield and composition of gasoline range oil. Biomass Convers Biorefin 2023; 13:13–29. https://doi.org/10.1007/s13399-020-01149-3.

[67] Bu Q, Liu Y, Liang J, Morgan HM, Yan L, Xu F, et al. Microwave-assisted co-pyrolysis of microwave torrefied biomass with waste plastics using ZSM-5 as a catalyst for high quality bio-oil. J Anal Appl Pyrolysis 2018; 134:536–43. https://doi.org/10.1016/j.jaap.2018.07.021.

[68] Bu Q, Chen K, Xie W, Liu Y, Cao M, Kong X, et al. Hydrocarbon rich bio-oil production, thermal behavior analysis and kinetic study of microwave-assisted co-pyrolysis of microwave-torrefied lignin with low density polyethylene. Bioresour Technol 2019;291. https://doi.org/10.1016/j.biortech.2019.121860.

[69] Lam SS, Wan Mahari WA, Ok YS, Peng W, Chong CT, Ma NL, et al. Microwave vacuum pyrolysis of waste plastic and used cooking oil for simultaneous waste reduction and sustainable energy conversion: Recovery of cleaner liquid fuel and techno-economic analysis. Renewable and Sustainable Energy Reviews 2019;115. https://doi.org/10.1016/j.rser.2019.109359.

[70] Wan Mahari WA, Chong CT, Cheng CK, Lee CL, Hendrata K, Yuh Yek PN, et al. Production of value-added liquid fuel via microwave co-pyrolysis of used frying oil and plastic waste. Energy 2018; 162:309–17. https://doi.org/10.1016/j.energy.2018.08.002.

[71] Saifuddin N, Priatharsini P, Hakim SB. Microwave-assisted co-pyrolysis of Bamboo biomass with plastic waste for hydrogen-rich syngas production. Am J Appl Sci 2016; 13:511–21. https://doi.org/10.3844/ajassp.2016.511.521.

[72] Zhang X, Yu Z, Lu X, Ma X. Catalytic co-pyrolysis of microwave pretreated chili straw and polypropylene to produce hydrocarbons-rich bio-oil. Bioresour. Technol. 2021;319. https://doi.org/10.1016/j.biortech.2020.124191.

[73] Zhao Y, Wang Y, Duan D, Ruan R, Fan L, Zhou Y, et al. Fast microwave-assisted ex-catalytic co-pyrolysis of bamboo and polypropylene for bio-oil production. Bioresour Technol. 2018; 249:69–75. https://doi.org/10.1016/j.biortech.2017.09.184.

[74] Ding K, Liu S, Huang Y, Liu S, Zhou N, Peng P, et al. Catalytic microwave-assisted pyrolysis of plastic waste over NiO and HY for gasoline-range hydrocarbons production. Energy Convers Manag 2019; 196:1316–25. https://doi.org/10.1016/j.enconman.2019.07.001.

[75] Monzavi M, Chen Z, Hussain A, Chaouki J. High quality products from microwave catalytic pyrolysis of heavy oil and polyethylene. Appl Therm Eng 2023;230. https://doi.org/10.1016/j.applthermaleng.2023.120722.

[76] Budsaereechai S, Hunt AJ, Ngernyen Y. Catalytic pyrolysis of plastic waste for the production of liquid fuels for engines. RSC Adv 2019; 9:5844–57. https://doi.org/10.1039/c8ra10058f.

[77] Zhang B, Zhong Z, Chen P, Ruan R. Microwave-assisted catalytic fast pyrolysis of biomass for bio-oil production using chemical vapor deposition modified HZSM-5 catalyst. Bioresour Technol 2015; 197:79–84. https://doi.org/10.1016/j.biortech.2015.08.063.

[78] Juliastuti SR, Hendrianie N, Ramadhan PJ, Satria DH. Microwave pyrolysis of multilayer plastic waste (LDPE) using zeolite catalyst. AIP Conf Proc, vol. 1840, American Institute of Physics Inc.; 2017. https://doi.org/10.1063/1.4982331.

[79] Fan L, Zhang Y, Liu S, Zhou N, Chen P, Liu Y, et al. Ex-situ catalytic upgrading of vapors from microwave-assisted pyrolysis of low-density polyethylene with MgO. Energy Convers Manag 2017; 149:432–41. https://doi.org/10.1016/j.enconman.2017.07.039.

[80] Tuli V, Luo C, Robinson B, Hu J, Wang Y. Microwave-assisted catalytic technology for sustainable production of valuable chemicals from plastic waste with enhanced catalyst reusability. Chemical Engineering Journal 2024;489. https://doi.org/10.1016/j.cej.2024.151551.

[81] Jing X, Dong J, Huang H, Deng Y, Wen H, Xu Z, et al. Interaction between feedstocks, absorbers and catalysts in the microwave pyrolysis process of waste plastics. J Clean Prod 2021;291. https://doi.org/10.1016/j.jclepro.2021.125857.

[82] Zhou N, Dai L, Lyu Y, Li H, Deng W, Guo F, et al. Catalytic pyrolysis of plastic wastes in a continuous microwave assisted pyrolysis system for fuel production. Chemical Engineering Journal 2021;418. https://doi.org/10.1016/j.cej.2021.129412.

[83] Shoukat B, Naz MY, Yaseen M, Noreen S. Microwave-Driven Pyrolysis of Plastic Waste into Carbon Nanotubes and Hydrogen Using Spinel Ferrites. Chem Eng Technol 2024; 47:1013–23. https://doi.org/10.1002/ceat.202300288.

[84] Ramzan F, Shoukat B, Naz MY, Shukrullah S, Ahmad F, Naz I, et al. Single step microwaves assisted catalytic conversion of plastic waste into valuable fuel and carbon nanotubes. Thermochim Acta 2022;715. https://doi.org/10.1016/j.tca.2022.179294.

[85] Wang J, Pan Y, Song J, Huang Q. A high-quality hydrogen production strategy from waste plastics through microwave-assisted reactions with heterogeneous bimetallic

iron/nickel/cerium catalysts. J Anal Appl Pyrolysis 2022; 166:105612. https://doi.org/10.1016/j.jaap.2022.105612.

[86] Fan S, Zhang Y, Cui L, Maqsood T, Nižetić S. Cleaner production of aviation oil from microwave-assisted pyrolysis of plastic wastes. J Clean Prod 2023;390. https://doi.org/10.1016/j.jclepro.2023.136102.

[87] Zhang X, Lei H, Yadavalli G, Zhu L, Wei Y, Liu Y. Gasoline-range hydrocarbons produced from microwave-induced pyrolysis of low-density polyethylene over ZSM-5. Fuel 2015; 144:33–42. https://doi.org/10.1016/j.fuel.2014.12.013.

[88] Hussain Z, Khan KM, Perveen S, Hussain K, Voelter W. The conversion of waste polystyrene into useful hydrocarbons by microwave-metal interaction pyrolysis. Fuel Processing Technology 2012; 94:145–50. https://doi.org/10.1016/j.fuproc.2011.10.009.
